# Interleukin-6 stimulates platelet 12-lipoxygenase to drive coagulation in inflammatory arthritis

**DOI:** 10.1016/j.jlr.2026.101093

**Published:** 2026-06-29

**Authors:** Daniela O. Costa, Stuart T.O. Hughes, Robert H. Jenkins, Ana Cardus Figueras, Majd B. Protty, Victoria J. Tyrrell, Ali A. Hajeyah, Gareth W. Jones, James J. Burston, Bethan Morgan, Federica Monaco, David Hill, Aisling S. Morrin, Carol Guy, Alice Bacon, Martin Giera, Rene E.M. Toes, P. Vince Jenkins, Peter W. Collins, Ernest Choy, Simon A. Jones, Valerie B. O’Donnell

**Affiliations:** 1 Systems Immunity Research Institute, Division of Infection and Immunity, School of Medicine, Cardiff University, Cardiff, UK; 2 School of Cellular and Molecular Medicine, University of Bristol, Bristol, United Kingdom; 3 Department of Rheumatology, Leiden University Medical Center, Leiden, The Netherlands; 4 Center for Proteomics and Metabolomics, Leiden University Medical Center, Leiden, The Netherlands; 5 Haematology Department, Cardiff and Vale University Health Board, Cardiff, United Kingdom

**Keywords:** arthritis, lipids, lipoxygenase, thrombosis, coagulation, inflammation, platelets

## Abstract

Rheumatoid arthritis (RA) is associated with significantly higher thrombotic risk, which is not mechanistically understood. In particular, the detailed mechanisms driving coagulation in arthritis have not been explored, despite the significant impact on patient morbidity and mortality. The role of platelet and blood cell enzymatically oxidized phospholipids (eoxPL) in driving thrombosis and their regulation by inflammation was tested using a mouse model antigen-induced arthritis (AIA) and a human RA cohort. AIA induction significantly elevated plasma thrombin-antithrombin (TAT) complexes, soluble P-selectin (sP-selectin) and serum amyloid A, indicating inflammation and elevated coagulation. Concurrently, platelet-derived eoxPL and their IgG autoantibodies were elevated in whole blood and plasma. These indices were all suppressed by genetic deletion of IL-6Ra, while platelet *Alox12* deletion prevented TAT, sP-selectin, and eoxPL/autoantibody increases. Leukocyte *Alox15* deletion did not impact circulating TATs or eoxPL. This indicates that IL-6 drives coagulation in AIA via upregulation of platelet *Alox12*. Conversely, synovial tissue demonstrated raised eoxPL exclusively from *Alox15*. In human RA, immunoreactivity towards platelet-derived eoxPL was elevated in early disease. Thus, while both LOX isoforms differentially contribute to AIA in mice, only *Alox12* promotes thrombosis risk, while evidence for increased eoxPL exposure in early human RA was revealed. In summary, the IL-6/*Alox12* axis drives platelet activation in AIA, providing a potential mechanistic explanation for recent clinical trial data proposing that anti-iL-6 therapy is associated with reduced coagulation in early RA. The procoagulant lipid membrane is proposed as a therapeutic target for preventing vascular events in autoimmune disease.

Rheumatoid arthritis (RA) is associated with increased thrombotic risk, evidenced by 30%–50% increased incidence of cardiovascular disease and stroke, yet it is currently not routinely monitored in patients (1, 2, 3, 4). Although mechanisms are unknown, a recent clinical trial showed that patients with RA have elevated coagulation markers which can be restored using anti-IL-6R (tociluzimab) (5), indicating that inflammation is involved. Coagulation is driven by circulating plasma factors which require an electronegative membrane provided by platelets and white blood cells, following agonist activation. This comprises phospholipids (PLs), namely phosphatidylserine (PS), phosphatidylethanolamine (PE), and enzymatically-oxidized phospholipids (eoxPL). These are either rapidly externalized (PS and PE) (6, 7) or generated de novo (eoxPL) following agonist activation (8, 9, 10). EoxPL are formed via esterification of oxylipins generated by either lipoxygenases (LOX) or cyclooxygenases (COX) or direct oxygenation of PL or lysoPL (10, 11).

The most important PL driving coagulation is PS, which through its association with calcium, supports binding of coagulation factors at the membrane surface. PE and/or eoxPL enhance the ability of PS to support coagulation factor binding and activation. In the case of eoxPL, this appears to be most likely due to biophysical changes that could include membrane flattening/thinning and/or increasing distance between PL headgroups (9, 10, 12).

Humans with antiphospholipid syndrome (APS), an autoimmune disease with elevated thrombotic risk, display higher levels of circulating eoxPL and their IgG autoantibodies (12), while eoxPL are also raised in atherosclerotic cardiovascular disease (13). This led us to ask whether the procoagulant PL membrane is increased in other immune-mediated inflammatory diseases such as RA and directly contributes to coagulation activity. Several thrombotic markers are increased in patients with RA, although there is limited understanding of the underlying mechanisms (14). For example, elevated thrombin activation, reflected by increased thrombin-antithrombin (TAT) complexes (15), soluble P-selectin (sP-selectin) and D-dimers have been described in RA plasma (16). Tissue factor (TF) positively correlates with plasma C-reactive protein and leukocyte counts, suggesting TF may play a role, and that thrombosis is associated with inflammation (15). However, prothrombin and thrombin times are normal in RA, indicating that coagulation factor levels are not increased (17). This indicates that other processes are involved, with the prothrombotic membrane being a strong candidate. Only a few studies have examined thrombotic risk in murine arthritis, and no mechanistic research has yet been performed (18). To address this information gap, studies in human cohorts and mouse models were undertaken, focusing on the role of the procoagulant eoxPL membrane. Our study demonstrates that eoxPL from platelet 12-LOX drive the coagulation phenotype in arthritis, in an IL-6-dependent manner. These findings provide mechanistic insight into recent trial data in humans and identify a new therapeutic target for lowering thrombotic risk in immune-mediated inflammatory diseases.

## Materials and methods

### Animal experiments

*Il27ra*^*−/−*^ (19), *Il6ra*^*−/−*^ (20), *Alox15*^*−/−*^, and *Alox12*^*−/−*^ (21) mice on C57BL/6 background were bred under specific pathogen-free conditions. Experiments were conducted in accordance with the United Kingdom Home Office Animals (Scientific Procedures) Act 1986, under project licenses PE8BCF782 and PC0174E40. antigen-induced arthritis (AIA) was induced (male, 9–12 weeks) as described (20), with full detail in Supplementary Methods. Whole joints were obtained and processed as outlined in Supplementary Methods for histology. Synovial tissue was dissected from the joint cavity and lipid extraction performed as in Supplementary Methods. Mouse blood collection and processing for analysis of inflammatory and coagulation parameters was performed as described (22), with full detail in Supplementary Methods. Lipid extraction of whole blood cells, acid hydrolysis, and LC-MS/MS analysis were conducted as in Supplementary Methods. *IL27ra*^*−/−*^ (19), IL*6ra*^*−/−*^ (20), *IL6*^*−/−*^ (23), *Alox15*^*−/−*^ (21), and *Alox12*^*−/−*^ (24) mice on C57BL/6 background were bred under specific pathogen-free conditions at Cardiff University (Cardiff, Wales). C57BL/6 WT mice were obtained from Charles Rivers Laboratories (United Kingdom) and were acclimatized for 2-weeks prior to experiments. Mice were housed in filter top cages (or scantainers, *IL6ra*^*−/−*^), with 12-h light/dark cycles at 20–22°C, and fed standard chow with unrestricted water. For tissue harvesting, mice were killed via CO_2_ inhalation, followed by cardiac puncture. All animal experiments were conducted in accordance with the United Kingdom Home Office Animals (Scientific Procedures) Act of 1986, under the project licenses PE8BCF782 and PC0174E40. Simple randomization was performed at cage level to allocate mice to the AIA model. AIA was induced in 9–12-week-old male mice, to minimize potential confounders, as previously described (25). For most measures, tissue/blood from one mouse represents an experimental unit. For lipid analysis of synovial tissue, pooling of joint tissue from more than one was necessary, ranging from 3 mice (6 joints) to 6 mice (12 joints). In brief, mice were subcutaneously injected with 100 μl mBSA/Complete Freund’s Adjuvant (Sigma-Aldrich) stable emulsion. Next, 100 μl *Bordetella Pertussis* toxin (1.6 ng/μl) was administered intraperitoneally. After one week, the mice were reimmunized with mBSA/Complete Freund’s Adjuvant on the opposite flank. 21 days after the first immunization, inflammatory arthritis was induced using an intra-articular (i.a.) injection of 10 μl mBSA (10 mg/ml). Both i.p, s.c., and i.a. injections were performed in a simple randomized order, with the researcher blinded to genotypes. Arthritis progression was monitored by measuring knee joint swelling with a POCO 2T micrometer (Kroeplin) in the morning to reduce confounding factors. Three days after arthritis triggering, mice were culled for the acute disease time point, while the chronic time point was 10 days post i.a. injection. Scoring criteria are shown in Supplemental Table S1. The sample size was initially determined based on data from a previous study (26), using an online sample size calculator^4^ and employing TAT complexes as the primary outcome measure. Each study group was determined to require at least 8 mice. However, it was possible to reduce this number to 6 and 7 in the case of *Il6ra*^*−/−*^ and *Alox12*^*−/−*^ mice, respectively, since a significant difference in the primary outcome measure, TAT complexes, was observed following the first experimental group test. A total of 181 mice were used across all experiments. Animals were observed daily and monitored for signs of distress. Only males were used since females show reduced incidence and a more variable phenotype in the model.

### LC/MS/MS analysis of eoxPLs

Lipid extracts were separated using reverse-phase HPLC on a Luna C_18_ column (150 mm × 2 mm × 3 μm) (Phenomenex, Torrance, CA). A gradient elution method of 50%–100% B over 10 min followed by 30 min at 100% B (A, methanol:acetonitrile:water, 1 mM NH_4_CH_3_CO_2_, 60:20:20; B, methanol, and 1 mM NH_4_CH_3_CO_2_) was applied with a total flow rate of 200 μl/min. Products were analyzed in multiple reaction monitoring (MRM) mode, on a 6500 Q-Trap (Sciex, Cheshire, United Kingdom), operating in the negative mode, using the following ion source parameters: Temperature: 500°C, Curtain gas (CUR): 35 psi, Source Gas 1 (GS1): 40 psi, Source Gas 2 (GS2): 30 psi, Ion spray voltage: −4500 V, entrance potential (EP): − 10 V, collision energy −38 V, declustering energy −50 V, collision cell exit potential −11 V. Transitions were monitored from precursor mass (Q1 *m/z*) to product ion mass (Q3 *m/z*), with a dwell time of 75 msec. For quantification, a mixed isomer HETE-PLs standard curve was generated and a known isomer ratio was used for the determination of each lipid isomer concentration (27). MRMs used are provided in Supplemental Table S2. LOD and LOQ were set at 3, and 5:1 respectively (S/N) with at least 6 data points per peak required. HETE-PLs were generated as mixed isomers, and used either as a racemic mixture or as individual positional isomers once isolated and purified, as previously described (27).

### LC/MS/MS analysis of oxylipins

Lipids were separated using reverse phase HPLC on an Agilent Eclipse Plus C_18_ column (150 mm × 2.1 mm × 1.8 μm) (Phenomenex, Torrance, CA) t 45°C, with a flow rate of 500 μl/min. A gradient elution method was used where mobile phase B is held at 30% for 1 min, then increased to 100% B from 1 - 17.5 min (A: 94.9% water, 5% solvent B, 0.1% glacial acetic acid; B: 84% acetonitrile, 15.9% methanol, and 0.1% glacial acetic acid), 100% B is held from 17.5 -21 min, followed by a decrease to 30% of B from 21–22.5 min, which is held until the end of the run at 22.5 min. Lipids were analyzed using a scheduled MRM method on a 6500 Q-Trap (Sciex, Cheshire, United Kingdom). A time window is set for the detection of each analyte according to the expected retention time, and transitions are monitored from precursor mass (Q1 *m/z*) to product ion mass (Q3 *m/z*) in negative ion mode, under the following ion source parameters: Temperature: 475°C, CUR: 35 psi, GS1: 60 psi, GS2: 60 psi, Ion spray voltage: −4500 V, EP: − 10V. The area under the curve for the precursor ion to product ion transition was integrated using Multiquant 3.0.2. (AB Sciex, Canada) and normalized to the corresponding IS. For quantification, specific isomeric standards were used to generate a standard curve. An equation for calculation was obtained using 1/x^2^ weighted linear regression. MRMs used are provided in Supplemental Table S3. LOD and LOQ were set at 3, and 5:1 respectively (S/N) with at least 6 data points per peak required.

### Alkaline hydrolysis of lipid extracts for chiral HETE analysis

Whole blood cell pellet lipid extracts were dried under a stream of N_2_ and resuspended in 1.5 ml IPA. Alkaline hydrolysis was accomplished by the addition of 1.5 ml 1 M NaOH, followed by a 30 min incubation at 60°C in a dry bath incubator. Afterward, the extracts were acidified to pH 3.0 using 150 μl 1 M HCl before reextraction. Briefly, to each sample 3 ml hexane was added, followed by vortexing and centrifugation (1475 g, 5 min, 4°C). The top organic layer was recovered, and another 3 ml hexane was added to the remaining bottom layer followed by vortexing and centrifugation. The top layer was recovered and combined with the previous isolated organic layer. The IS used was 12(S)-HETE-d8, which was already present in the lipid extracts, as previously. The combined layers were dried using RapidVap Vacuum. Lipids were resuspended in 150 μl methanol, and stored at − 80°C in an N_2_ atmosphere until analysis by LC/MS/MS.

### Chiral LC/MS/MS

Separation was achieved using reversed-phase HPLC on a ChiralPak AD-RH column (150 mm × 4.6 mm × 5 μm; Daicel Corporation) with an isocratic gradient of methanol:water:glacial acetic acid 95:5:0.1 (v/v) with flow rate 300 μl/min for 25 min at 40°C. Products were analyzed in MRM mode, on a 4000 Q-Trap (Sciex, Cheshire, United Kingdom). Transitions were monitored from precursor mass (Q1 *m/z*) to product ion mass (Q3 *m/z*) in negative ion mode, with a dwell time of 125 msec, with the following ion source parameters: Temperature: 500°C, CUR: 20 psi, GS1: 40 psi, GS2: 30 psi, Ion spray voltage: −4500 V, EP: − 10V. MRMs used are provided in Supplemental Table S4. LOD and LOQ were set at 3, and 5:1 respectively (S/N) with at least 6 data points per peak required.

### Human studies

All human studies followed the principles of the Declaration of Helsinki, with informed consent and full ethical approval. Serum from RA patients was obtained from the Leiden Early Arthritis Clinic (Department of Rheumatology, Leiden University Medical Center) (28). The study was approved by the LUMC Biobank Ethical Review Committee, and all patients gave written informed consent. Samples of serum from healthy controls were obtained under the project “Study of the lipidomic profile of blood clots from healthy volunteer*s”* approved by the School of Medicine Ethics Review Committee, Cardiff University (REC/SREC reference No 16/02, study 10). Serum was obtained using a 21-gauge butterfly needle and a BD vacutainer® (Thermo Fisher Scientific, United Kingdom). Blood was drawn to a final volume of 10 ml. After mixing, the blood was allowed to clot, over 30 min. Serum was isolated after centrifugation (2,340 g, 10 min) and stored immediately at −80°C. The Leiden samples were compared with serum generated from blood obtained from healthy volunteers from Cardiff, United Kingdom since no healthy controls were available from Leiden. The blood draw and serum isolation process followed the same protocol for both sites. Clinical characteristics of these groups including when blood was sampled, disease activity, medication status, and sample size are given in Supplemental Table S5.

### Analysis of serum IgG against eoxPL in human RA samples

HETE-PE autoantibody titers were determined by chemiluminescent ELISA assay^3^. 5-HETE-PEs, 12-HETE-PEs, 15-HETE-PEs, 8-HETE-PEs, and 1-stearoyl-2-arachidonyl-PE (SAPE), were diluted to 20 μg/μl, and 25 μl added to a well of a PolySorp® surface plate (Thermo Fisher scientific), followed by drying under N_2_ stream. Each well was blocked using 0.5% (w/v) fish-gelatine in 0.27 mM EDTA/DPBS (55 μl) and incubated for 1 h. In each well, 50 μl serum, diluted (1:12) in DPBS-0.27 mM EDTA, was incubated for 90 min. Wells were washed 3 times with DPBS/EDTA solution, before adding 25 μl antihuman IgG alkaline phosphatase-conjugated secondary antibody (Sigma-Aldrich) diluted 1:20,000 in blocking solution. After another wash, 25 μl LumiPhos 530 (Lumigen, Inc), diluted 1:3 in H_2_O, was added to each well. Following incubation for 90 min, luminescence was read on a microplate reader (CLARIOstar Plus) and data expressed as relative light units/100 ms (RLU/100 ms).

### Statistical analysis

Statistical analysis used Graphpad Prism 9. Missing values below LOD were input as 50% of the lowest detected value. No statistical test was performed if more than 50% of data were missing. Shapiro-Wilk test was used for normality testing. Nonparametric analysis was performed using Mann-Whitney (pairs) or Kruskal–Wallis tests (more than 2 groups). Pairwise differences were determined using Dunn's multiple comparisons test. Where data were normally distributed, Student *t* test, one-way or two-way ANOVA was used with Tukey’s Post Hoc test. Data are shown comparing response to disease induction within individual genotypes only, for simplicity of comparison. Heatmaps and hierarchical clustering were used a Pheatmap package in R. Heatmaps display log10 of averaged lipid amounts (ng), normalized to cell count, volume (ml) or wet tissue weight (mg), allowing row-wise and column-wise comparison.

## Results

### TATs, sP-selectin and serum amyloid A (SAA) are increased in murine arthritis

The AIA model is widely used and well characterized as a surrogate for systemic inflammatory arthritis, showing similar features to human disease. For example, AIA closely matches transcriptional changes observed in synovial biopsies from patients with synovial pathologies (29). AIA was used for development of biological medicines in routine clinical practice as well as new therapies currently in trials (30, 31, 32, 33). AIA leads to systemic inflammation, in line with human RA, with this directly relevant to our study focusing on coagulation in the vasculature (26, 34, 35, 36). Additional information on the choice of model is in Supplementary Methods.

**Fig. 1:**
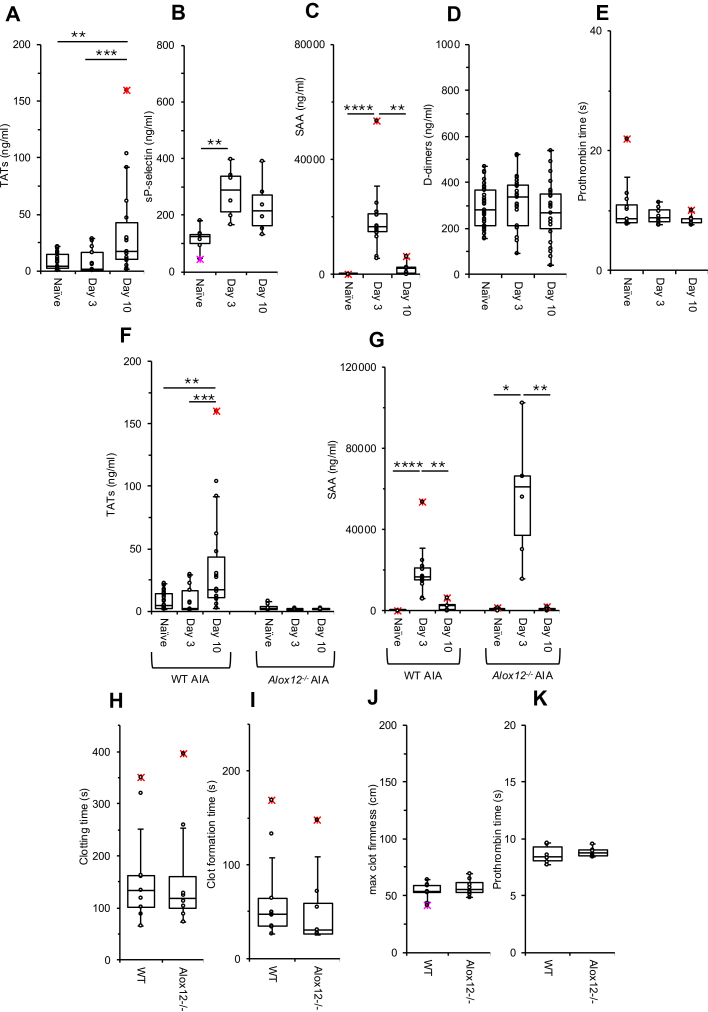
Murine arthritis increases coagulation and inflammation in WT but not Alox12^−/−^ mice, while coagulation factors themselves are not impacted in Alox12^−/−^. AIA was induced in 8–12-week-old male mice as described in Methods, with whole blood collected on days 3 and 10. Panel A: TATs are elevated on day 10 in AIA. TAT complexes were measured using ELISA. Plasma was collected from naïve mice, and on days 3 and 10 of AIA development from WT mice (n = 34, 19, and 18, respectively). Data were analyzed using Kruskal-Wallis test and Dunn’s multiple comparisons tests (∗∗*P* < 0.01, ∗∗∗*P* < 0.005). Panel B: soluble P-selectin is increased in AIA. sP-selectin was measured using ELISA. Plasma was collected from naïve mice, and on days 3 and 10 of AIA development from WT mice (n = 6 for all) mice. Data were analyzed using one-way ANOVA and Tukey’s multiple comparisons tests, (∗∗*P* < 0.01). Panel C: SAA is elevated in AIA. SAA was measured using ELISA. Plasma was collected from naive mice and on day 3 and 10 of AIA development from WT mice (n = 10, 14, and 11, respectively). Data were analyzed using Kruskal-Wallis test and Dunn’s multiple comparisons tests (∗∗*P* < 0.01, ∗∗∗∗*P* < 0.001). Panel D: AIA induction does not alter D-Dimer levels. D-dimers were measured using ELISA. Plasma was collected from naïve and on days 0, 3, and 10 of AIA development from WT mice (n = 33, 25, and 23, respectively). Data were analyzed using one-way ANOVA and Tukey’s multiple comparison test. Panel E: AIA induction does not alter prothrombin time. Prothrombin time was determined as described in Methods. Plasma was collected from naïve mice and on days 3 and 10 of AIA development in WT mice (n = 12, 8, and 9, respectively). Data were analyzed using the one-way ANOVA and Tukey’s multiple comparison test. Panel F: *Alox12* deletion prevents elevation of TAT complexes during AIA. TAT complexes were measured using ELISA. Plasma was collected from WT (n = 34) and *Alox12*^*−/−*^ naïve mice, as well as on days 3 and 10 of AIA development in WT (n *=* 19 and 18, respectively) and*, Alox12*^*−/−*^ (n = 7 for both days). Data were analyzed using Kruskal-Wallis test and Dunn’s multiple comparisons tests, comparing within genotypes (∗∗*P* < 0.01, ∗∗∗*P* < 0.001). Panel G: SAA elevation in AIA is not dependent on *Alox12*. SAA was measured using ELISA. Plasma was collected from WT (n = 9) or *Alox12*^*−/−*^ (n = 6) naïve mice, as well as on days 3 and 10 of AIA development in WT (n *=* 15 and 11, respectively) and *Alox12*^*−/−*^ (n = 6 and 7, respectively). Data were analyzed using Kruskal-Wallis test and Dunn’s multiple comparisons tests, comparing within genotypes (∗*P* < 0.05, ∗∗*P* < 0.01, ∗∗∗∗*P* < 0.001). Panel H-K: Basal coagulation is not altered in Alox12^−/−^ mice. ROTEM® data for male WT and *Alox12*^*−/−*^ mice aged 12–14 weeks (n = 9 and 8 respectively). (H) clotting time, (I) time to maximum clot firmness, and (J) maximum clot firmness determined by ROTEM of whole blood murine samples with addition of 0.6%(v/v) innovin and 10 mM CaCl2. Clotted samples were removed from analysis. (K) prothrombin time (PT) for murine plasma samples (n = 6 and 6, respectively). Clotted samples were removed from analysis. Statistics performed by Student *t* test. AIA, antigen-induced arthritis; SAA, serum amyloid A; TAT, thrombin-antithrombin.

First, coagulation activity was characterized during AIA development. Prior to initiating AIA, it was confirmed that immunization of mice (the first part of the model) without subsequent AIA induction did not impact TATs, d-dimers or prothrombin time (PT) (Supplemental Fig. S1, A–C). Next, coagulation parameters were determined in plasma on days 3 and 10 of AIA. TATs and sP-selectin significantly increased during AIA in WT mice (Fig. 1A, B). Importantly, TATs are a well-established marker of thrombin generation and are universally determined in clinical settings for assessment of acute blood clotting, as an early predictor of deep vein thrombosis and hypercoagulation (37, 38). To examine for inflammation, serum amyloid A (SAA) was measured and found to increase transiently in WT earlier than TATs (Fig. 1C). D-dimers or PT were not impacted at either timepoint (Fig. 1D, E), indicating that while activation of coagulation occurred, there was no increase in fibrin degradation or consumptive coagulopathy. This is consistent with elevated thrombotic risk and is similar to human RA, where TATs are elevated, but no alterations to PT or partial thromboplastin times are observed (17). Increased D-dimer has been reported in human RA (39), however the short timescale of the AIA model may be insufficient for fibrinolysis to have occurred.

### Deletion of Alox12 in AIA prevents thrombin activation, but not inflammation, while deletion of Alox15 has no impact

Next, to determine whether LOX isoforms contribute to thrombosis and inflammation, plasma TATs and SAA were determined in *Alox12*^*−/−*^ or *Alox15*^*−/−*^ mice. Platelet *Alox12* deletion totally prevented TAT increases on day 10 (Fig. 1F). In contrast, SAA was increased at day 3, similar to WT, indicating that inflammation is not dependent on *Alox12* (Fig. 1G). To check whether the increased TATs could have been driven by higher overall clotting factor levels, coagulation parameters were analyzed in blood from naïve mice. No differences between WT and *Alox12*^*−/−*^ were found, indicating that other mechanisms drive the phenotype (Fig. 1H–K). These include the *Alox12*-dependent procoagulant membrane as will be explored below. In contrast to *Alox12*, no role for the leukocyte type isoform *Alox15,* was found in driving TAT elevations or inflammation during AIA induction (Supplemental Fig. S1E, E).

### Circulating procoagulant eoxPL are increased in murine AIA and dependent on Alox12, but not Alox15

Coagulation initiated by TF requires a procoagulant membrane provided by circulating blood cells, primarily platelets. EoxPL are a central component of this, generated by blood cells following activation (40, 41), but whether they are elevated in AIA, and contribute to thrombotic risk is unknown. A panel of eoxPL including isomers generated by LOX or COX enzymes in platelets and white blood cells were measured in mouse blood cells using lipidomics. These include: 12-HETE-PEs (expressed in platelets, *Alox12*; expressed in leukocytes, *Alox15*), 11-HETE-PEs (expressed in platelets or leukocytes, COX-1/*Ptgs1*), 15-HETE-PEs (expressed in leukocytes, *Alox15*; expressed in platelets or leukocytes, *Ptgs1*), 5-HETE-PEs (expressed in leukocytes, *Alox5*), and 8-HETE-PEs (nonenzymatic or a minor product from *Alox12*) (42). First, it was confirmed that immunization alone of WT mice (prior to arthritis induction) did not significantly change blood cell eoxPL levels (Supplemental Fig. S1F–J). The most abundant detected were 12-HETE-PEs, including both plasmalogen and diacyl species with aliphatic chains 18:0, 18:1, or 16:0 at the *Sn1* position.

**Fig. 2:**
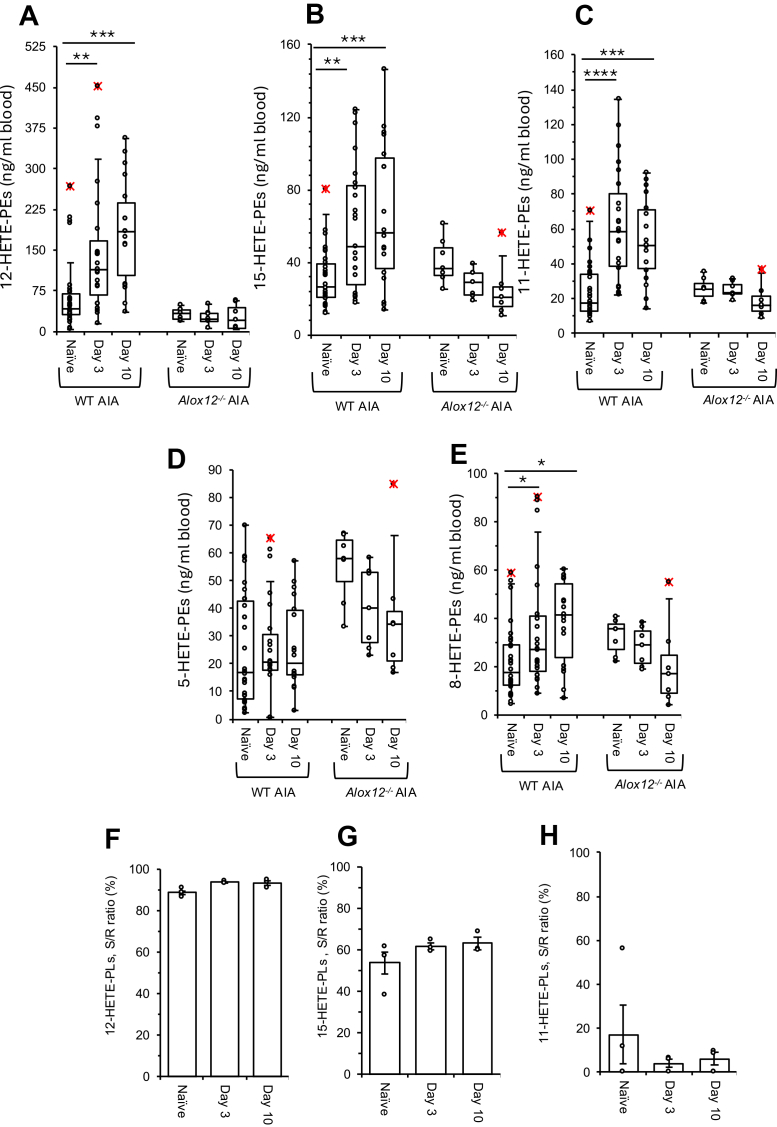
HETE-PEs levels increase in mouse blood cells during AIA development, and are dependent on *Alox12*. AIA was induced in 8–12-week-old *WT or Alox12*^−/−^ male mice as described in Methods, with whole blood collected on Days 3 and 10. Panel A-C: 12-, 15-, and 11-HETE-PEs were significantly elevated at day 10 of AIA, but not in *Alox12*^*−/−*^ mice. HETE-PL were quantified as outlined in Methods using LC/MS/MS. Whole blood was collected from WT naïve (n = 30) and *Alox12*^*−/−*^ naïve (n = 7) mice, as well as on days 3 and 10 of AIA development: WT (n = 23 and 18, respectively) and *Alox12*^*−/−*^ (n = 7 for both days). Data were analyzed using one-way ANOVA and Tukey’s multiple comparisons tests, comparing within genotypes (∗∗*P* < 0.01, ∗∗∗*P* < 0.001, ∗∗∗∗*P* < 0.001). Panels D and E: AIA increases 8-HETE-PEs in WT but not Alox12^−/−^ mice, but AIA has little impact on 5-HETE-PEs in either strain. Total 5-HETE-PEs and 8-HETE-PEs were determined in WT and *Alox12*^*−/−*^ mice, as outlined in Methods using LC/MS/MS with mouse numbers the same as Panels A–C. Data were analyzed using one-way ANOVA and Tukey’s multiple comparison test, comparing within genotypes (∗*P* < 0.05). Panel F-H: Increased generation of 12-, 15- and 11-HETE-PL during AIA is enzymatic. Chirality of eoxPL was determined in pooled lipid extracts as outlined in Methods using LC/MS/MS and expressed as S/R ratio (%) (n = 3) and represented by bar chart plot. AIA, antigen-induced arthritis; eoxPL, enzymatically oxidized phospholipid; HETE, hydroxyeicosatetraenoic acid; PE, phosphatidylethanolamine; PL, phospholipid.

Next, eoxPL generation AIA was determined in WT and platelet *Alox12*-deficient mice following AIA induction. Using LC/MS/MS, several individual isomers were determined, including with 16:0p, 18:0p, 18:1p, and 18:0a at *Sn-1*, and with either 5-, 8-, 11-, 12-, or 15-HETE at *Sn-2*, since these are the most abundant detected in blood cells (40, 41). Several HETE-PE isoforms, specifically 12-, 15-, and 11-HETE-PEs were elevated in blood cells from WT mice on days 3 and 10 (Fig. 2A–C, Supplemental Fig. S2A). 8-HETE-PEs showed a small increase while 5-HETE-PEs did not change (Fig. 2D, E). 12-, 15-, and 11-HETE-PEs did not increase in *Alox12*-deficient mice, overall indicating reduced platelet activation (Fig. 2A–C). The small increase in 8-HETE-PE was less apparent in *Alox12*-deficient mice, while 5-HETE-PE was generated at slightly higher levels (Fig. 2D, E). Overall, the data suggest that eoxPL from platelet 12-LOX and COX-1, but not leukocyte 5-LOX are significantly increased during induction of AIA. This is consistent with the timescale of TAT elevations which were not initiated during immunization but only on AIA induction.

To further confirm their enzymatic origin, HETE-PE stereoisomers were characterized using chiral chromatography. For this, lipid extracts were first saponified, then free HETEs analyzed for *S*- and *R*-enantiomer composition. More than 85% of 12-HETE was *S*-configuration, confirming generation by platelet 12-LOX (Fig. 2F). For 15-HETE, *S/R* ratio varied from 53% to 77%, depending on strain and disease stage (Fig. 2G). This isomer can be generated as *S*- or *R*-enantiomers by platelet COX-1 (43, 44). 11-HETE was < 20% *S*-enantiomer, consistent with generation by platelet COX-1 (Fig. 2H). Due to the low abundance of 8- and 5-HETEs, determining chirality of these lipids was not possible. Overall, this confirms that most abundant HETE-PEs generated during AIA are enzymatically generated, most likely via platelet 12-LOX and COX-1.

Generation of eoxPL in leukocyte-type *Alox15*-deficient mice was next explored during AIA induction since this isoform is expressed in circulating eosinophils and was previously shown to contribute to coagulation in mice (45). Here, AIA-induced eoxPL generation was not suppressed for either 5-, 12-, 11-, or 15-HETE-PEs, indicating they did not originate from leukocyte 12/15-LOX (Supplemental Fig. S2B–F). A small decrease in 5-HETE-PEs was noted, although total levels of these isoforms were far lower than for platelet isoforms. Overall, this indicates that 12/15-LOX is not contributing to the AIA-induced eoxPL elevation seen in WT blood cells.

### Increased coagulation activity and 12-LOX dependent eoxPL generation in AIA is dependent on IL-6, but not IL-27 signaling

Next, mouse strains lacking prominent cytokines that drive development of specific AIA pathotypes were investigated to determine the involvement of inflammation in promoting coagulation and eoxPL generation. During development of AIA, WT, *Il27ra*^−/−^, or *Il6ra*^*−/−*^ mice develop synovitis pathotypes that map to human arthritis pathotypes (29, 31, 34, 35, 46, 47). These include myeloid-rich disease, driven by macrophages (WT mice), lymphoid-rich disease, with an elevated adaptive immune response (*Il27ra*^−/−^), or a fibroblast-rich phenotype, with reduced immune inflammation (*Il6ra*^*−/−*^) (29, 31, 34, 35, 46, 47). First, induction of AIA was confirmed by joint swelling in all 3 mouse strains (Supplemental Fig. S3A). Platelet counts were normal for all 3 strains basally, and for WT and *Il6ra*^*−/−*^ mice, there was no impact of AIA (Supplemental Fig. S3B, C). Furthermore, all these strains have relatively normal blood cell counts (19, 21, 48, 49, 50).

**Fig. 3:**
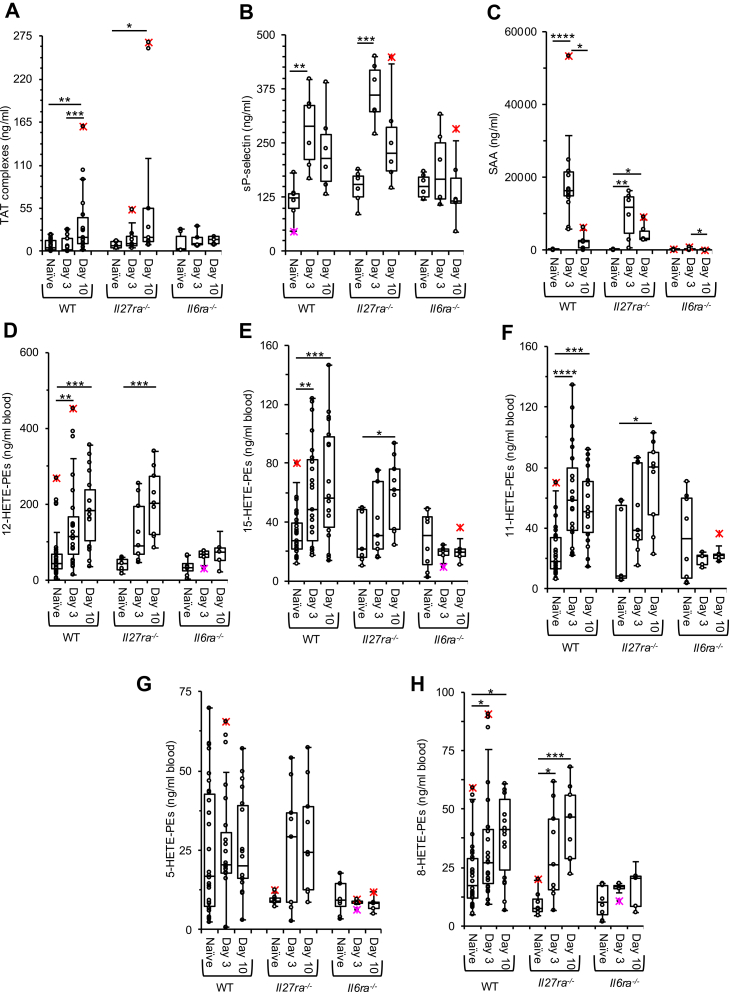
Murine arthritis increases TATs, sP-selectin, SAA, and platelet eoxPL in WT and *Il27ra*^−/−^ mice, but not *IL6ra*^−/−^. AIA was induced in 8–12-week-old *WT, Il27ra*^−/−^, and *Il6ra*^−/−^ male mice as described in Methods, with whole blood collected on days 3 and 10. Panel A: TATs are elevated on Day 10 in AIA, but not in IL6ra^−/−^ mice. TAT complexes were measured using ELISA. Plasma was collected from WT (n = 34), *Il27ra*^−/−^ (n = 7), and *Il6ra*^−/−^ (n = 10) naïve mice, as well as on days 3 and 10 of AIA development from WT (n *=* 19 and 18, respectively), *Il27ra*^−/−^ (n = 9), and *Il6ra*^−/−^ (n = 5). Data were analyzed using Kruskal-Wallis test and Dunn’s multiple comparisons tests, comparing within genotypes (∗*P* < 0.05, ∗∗*P* < 0.01, ∗∗∗*P* < 0.001). Panel B: soluble P-selectin is increased in AIA, but not in *IL6ra*^*−/−*^ mice. sP-selectin was measured using ELISA. Plasma was collected from WT (n = 6), *Il27ra*^−/−^ (n = 6), and *Il6ra*^−/−^ (n = 6) naïve mice, as well as on days 3 and 10 of AIA development from *WT (*n *= 6), Il27ra*^−/−^ (n = 6), and *Il6ra*^−/−^ (n = 6). Data were analyzed using one-way ANOVA and Tukey’s multiple comparisons tests, comparing within genotypes (∗∗*P* < 0.01,∗∗∗*P* < 0.001). Panel C: SAA is elevated in AIA, but not in Il6ra^−/−^ mice. SAA was measured using ELISA. Plasma was collected from WT (n = 10), *Il27ra*^−/−^ (n = 7), and *Il6ra*^−/−^ (n = 9) naïve mice, as well as on days 3 and 10 of AIA development from WT (n *=* 14 and 11, respectively), *Il27ra*^−/−^ (n *= 6* for both days), and *Il6ra*^−/−^ (n = 6 for both days). Data were analyzed using Kruskal-Wallis test and Dunn’s multiple comparisons tests, comparing within genotypes (∗*P* < 0.05, ∗∗*P* < 0.01,∗∗∗∗*P* < 0.0001). Panel D-F: 12-, 15-, and 11-HETE-PEs were significantly elevated in AIA, but not in IL6ra^−/−^ mice. HETE-PL were quantified as outlined in Methods using LC/MS/MS. Whole blood was collected from WT (n = 30), *Il27ra*^*−/−*^ (n = 7), and *Il6ra*^*−/−*^ (n = 8) naïve mice, as well as, on days 3 and 10 of AIA development *WT* (n = 23 and 18, respectively), *Il27ra*^−/−^ (n = 9 for both days), and *Il6ra*^−/−^ (n = 5 for both days). Data were analyzed using one-way ANOVA and Tukey’s multiple comparisons tests, comparing within genotypes (∗*P* < 0.05, ∗∗*P* < 0.01, ∗∗∗*P* < 0.001, ∗∗∗∗*P* < 0.0001). Panels G and H: 8-HETE-PEs increase during development of AIA, but not in Il6ra^−/−^ mice, while 5-HETE-PEs remain similar throughout. The sum of 8- and 5-HETE-PEs were quantified using the same mice as for Panels D–F, as outlined in Methods using LC/MS/MS. Data were analyzed using one-way ANOVA and Tukey’s multiple comparison test, comparing within genotypes (∗*P* < 0.05, ∗∗∗*P* < 0.001). AIA, antigen-induced arthritis; eoxPL, enzymatically oxidized phospholipid; HETE, hydroxyeicosatetraenoic acid; PE, phosphatidyethanolamine; SAA, serum amyloid A; sP-selectin, soluble P-selectin; TAT, thrombin-antithrombin.

Next, coagulation parameters were determined in plasma on days 3 and 10 of AIA. TATs and sP-selectin significantly increased during AIA in *Il27ra*^*−/−*^, as in WT, but not in *Il6ra*^*−/−*^ mice (Fig. 3A, B). This indicates that coagulation is stimulated by IL-6-driven pathways in AIA. As for WT, D-dimers or PT were not impacted at either timepoint in these strains (Supplemental Fig. S3D, E). Similar to WT, SAA increased in *Il27ra*^*−/−*^ earlier than TATs, but there was no increase in *Il6ra*^*−/−*^ mice (Fig. 3C), consistent with the known induction of SAA by IL-6 (51) and its use as a biomarker of venous thromboembolism in humans (52). Overall, these data show that IL-6 signaling, but not IL-27, is required for both inflammation and coagulation activity during induction of AIA.

Next, generation of eoxPL generation was compared. 12-, 15-, and 11-HETE-PEs were similarly elevated in blood cells from WT and *Il27ra*^*−/−*^ mice during AIA development, especially at day 10, while they were not elevated in *Il6ra*^*−/−*^ mice at either timepoint (Fig. 3D–H). This was mainly driven by 12-HETE-PE increases, with the same pattern seen for all individual groups of positional isomers (Supplemental Fig. S3F). Taken together, these data indicate that IL-6-driven inflammation is required for upregulation of the procoagulant membrane and coagulation activity during AIA development.

### Platelet derived oxylipins from 12-LOX are elevated by IL-6 signaling in AIA

**Fig. 4:**
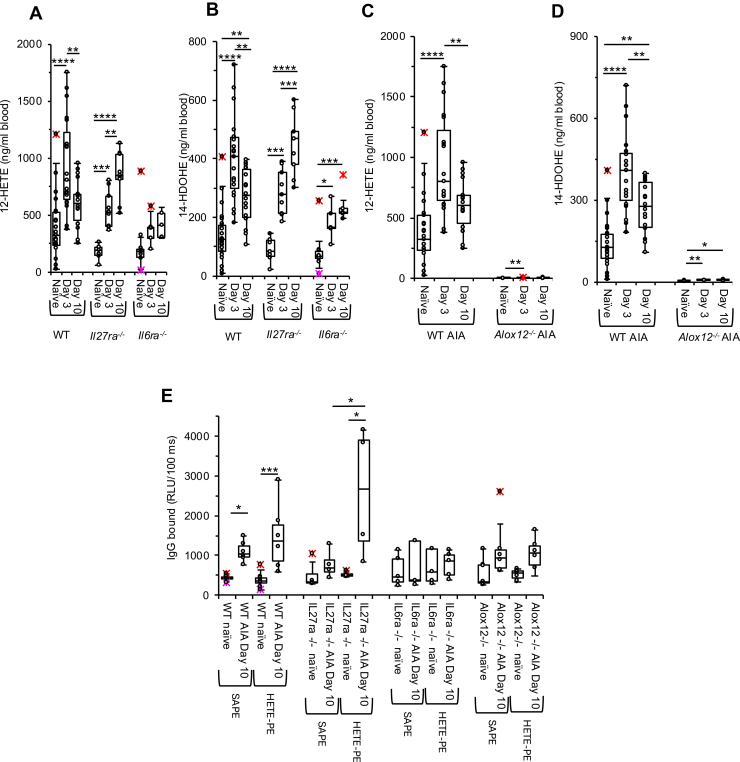
*IL6ra* or *Alox12* deletion prevents formation of platelet 12-LOX oxylipins and elevation of anti-HETE-PE IgG during AIA. AIA was induced in 8–12-week-old male mice as described in Methods. Panels A and B: 12-HETE and 14-HDOHE formation during AIA in WT, Il27ra^−/−^ and Il6ra^−/−^ mice. Whole blood was collected from WT (n = 25), *Il27ra*^−/−^ (n = 7), and *Il6ra*^−/−^ (n = 10) naïve mice, as well as on days 3 and 10 of AIA development in *WT* (n = 19 for day 3; n = 18 for day 10), *Il27ra*^−/−^ (n = 9 for both days), and *Il6ra*^−/−^ (n = 5 for both days) mice. Oxylipins were quantified as outlined in Methods using LC/MS/MS. Data were analyzed using one-way ANOVA and Tukey’s multiple comparison test within genotypes (∗*P* < 0.05, ∗∗*P* < 0.01, ∗∗∗*P* < 0.001, ∗∗∗∗*P* < 0.0001). Panels C and D: 12-HETE and 14-HDOHE formation during AIA in WT and *Alox12*^*−/−*^ mice. Whole blood was collected from WT (n = 25), and *Alox12*^−/−^ (n = 5) naïve mice, as well as, on day 3 and 10 of AIA development in *WT* (n = 19 for day 3; n = 18 for day 10), and *Alox12*^−/−^ (n = 7 for both days). Oxylipins were quantified as outlined in Methods using LC/MS/MS. Data were analyzed using one-way ANOVA and Tukey’s multiple comparison test within genotypes (∗*P* < 0.05, ∗∗*P* < 0.01, ∗∗∗∗*P* < 0.0001). Panel E: Anti HETE-PE IgG is significantly elevated at day 10 of AIA in WT and IL27ra^−/−^, but not in Il6ra^−/−^ or *Alox12*^*−/−*^ mice. Plasma was collected from WT (n = 9), *Il27ra*^−/−^ (n = 4), *Il6ra*^−/−^ (n = 5), and *Alox12*^−/−^ (n = 7) naïve mice, as well as on Day 10: WT (n = 6), *Il27ra*^−/−^ (n = 4), *Il6ra*^−/−^ (n = 5), and *Alox12*^−/−^ (n = 6). IgG levels against 15-HETE-PE were quantified as outlined in Methods and compared to IgG levels against SAPE. Data were analyzed using two-way ANOVA and Tukey’s multiple comparisons tests, comparing within genotypes (∗*P* < 0.05, ∗∗∗*P* < 0.001). AIA, antigen-induced arthritis; HDOHE, hydroxydocosahexaenoic acid; HETE, hydroxyeicosatetraenoic acid; LOX, lipoxygenase; PE, phosphatidylethanolamine; SAPE, 1-stearoyl-2-arachidonyl.

Next, free oxylipins were analyzed in blood cells isolated from mice during development of AIA. The data showed a 12-LOX activation signature on days 3 and 10, dominated by strong elevations in 12-HETE and 14-HDOHE, which were dependent on both IL-6 and platelet 12-LOX, but did not require IL-27 signaling (Fig. 4A–D). This further implicates IL-6 in driving platelet activation in AIA with the complete oxylipin datasets shown in full in Supplemental Figs. S4 and S5.

### AIA increases anti-eoxPL IgG, via both IL-6 and platelet 12-LOX

OxPL generation is associated with development of autoimmunity in other murine vascular inflammatory models, and we previously showed that humans with APS have elevated levels of eoxPL and higher anti-IgG eoxPL in their circulation (12, 53). Thus, we next examined whether the increased ability of platelets to generate eoxPL in AIA was associated with development of autoimmunity in this model. At day 10, WT and *Il27ra*^*−/−*^ mice both showed significantly increased immunoreactivity towards both 15-HETE-PE and its unoxidized isomer, SAPE, with considerably higher and more variable responses to the eoxPL (Fig. 4E). This response was absent in *Il6ra*^*−/−*^ and *Alox12*^*−/−*^ mice indicating it was IL-6 and platelet 12-LOX dependent. The lack of autoantibody response during AIA is fully consistent with the lack of elevation of eoxPL generation shown previously for these strains.

### AIA induction is not impacted by Alox15 or Alox12 deletion

**Fig. 5:**
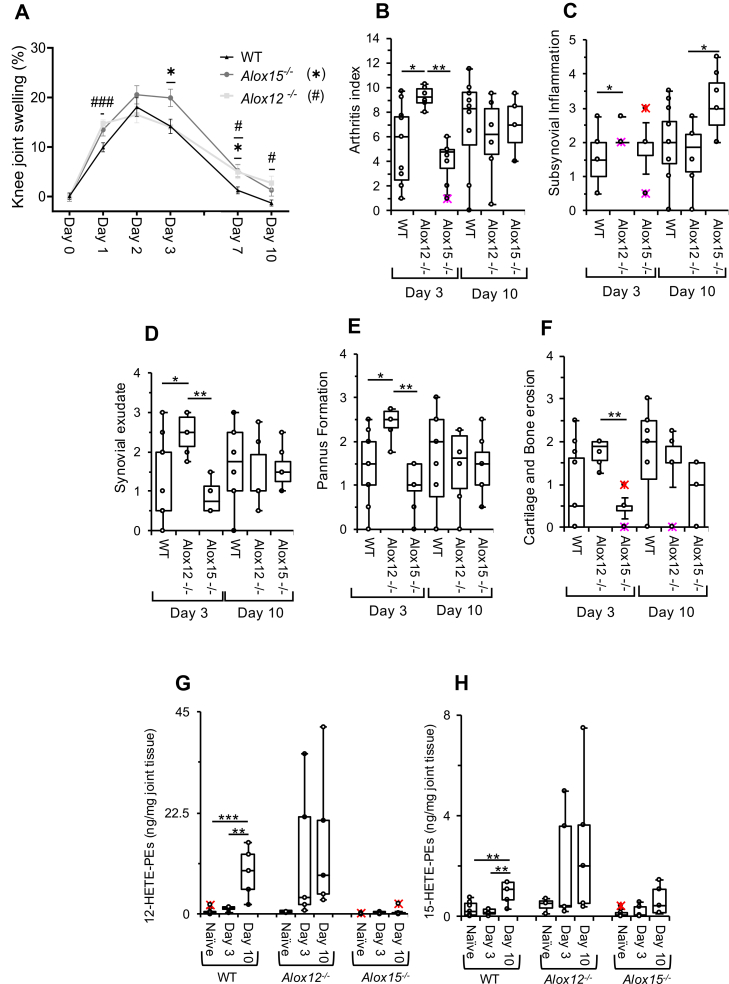
*Alox15*^*−/−*^*and Alox12*^*−/−*^ mice display a more severe AIA phenotype than WT, while *Alox15*^*−/−*^ display an overall reduction of eoxPL in synovial tissue during AIA development. AIA was induced in 8–12-week-old WT, *Alox12*^−/−^, and *Alox15*^−/−^ male mice as described in Methods, with synovial tissue collected on days 3 and 10. Panel A: Joint swelling is increased upon *Alox12* and *Alox15* deletion. Knee diameter was measured every 1–2 days and knee joint swelling was calculated as a percentage relative to knee diameter at day 0 (before arthritis induction) in WT (n = 44), *Alox15*^*−/−*^ (n = 32) and *Alox12*^*−/−*^ mice (n = 14). Data represent SEM, and statistical analysis ,was performed using a two-way ANOVA and Tukey multiple comparison test, comparing, on different days of AIA development, WT with *Alox15*^*−/−*^ (∗*P* < 0.05) and WT with *Alox12*^*−/−*^ mice (#*P* < 0.05, ###*P* < 0.001). Panels B-F: Arthritis index in AIA is more severe upon *Alox12* deletion. Knee joints were collected on day 3 and 10 from WT (n = 11 for day 3; n = 12 for day 10), *Alox12*^*−/−*^ (n = 6), and *Alox15*^*−/−*^ mice (n = 8 for day 3 and n = 7 for day 10) of AIA for histological staining and assessment, as described in Methods. Histopathology scoring of AIA was used to obtain Arthritis index, subsynovial inflammation, synovial exudate, pannus formation, and cartilage and bone erosion. Data were analyzed using one-way ANOVA test and Tukey’s multiple comparisons test, comparing within genotypes (∗*P* < 0.05, ∗∗*P* < 0.01). Panels G and H: HETE-PE generation in the synovial tissue during AIA is dependent on *Alox15*. 12- and 15-HETE-PEs, analyzed using LC/MS/MS, as outlined in Methods are shown. Multiple joints were collected and pooled from WT (n = 6, 6 joints/n), *Alox12*^*−/−*^(n = 3, 6 joints/n), and *Alox15*^*−/−*^ (n = 5, 6 joints/n) naïve mice, as well as on days 3 and 10 of AIA development (n *= 5,* 3–4 joints/n). Data were analyzed using one-way ANOVA and Tukey’s multiple comparison test, comparing within genotypes *(*∗∗*P* < 0.01, ∗∗∗*P* < 0.001). AIA, antigen-induced arthritis; HEPE, hydroxyeicosatetraenoic acid; PE, phosphatidylethanolamine.

Next, the role of *Alox15* and *Alox12* in joint pathology was determined. First, antibody titers to mBSA were not altered by deletion of either LOX, indicating that disease induction was normal (Supplemental Fig. S6A). Both strains showed a slightly more rapid development of joint swelling (on day 1) that resolved more slowly than WT by day 10 (Fig. 5A). Although significantly different, the overall pattern of joint disease appeared similar for all 3 strains. Next, joints were histologically scored for arthritis index. Deletion of *Al*ox*12* resulted in a worse phenotype at day 3, while *Alox15*^*−/−*^ mice were not different to WT (Fig. 5B, Supplemental Fig. S6). In contrast, in *Alox15*^*−/−*^ mice, a significant increase in subsynovial inflammation was observed at day 10 compared to *Alox12*^*−/−*^ (Fig. 5C). On day 3, synovial exudate and pannus formation scores in *Alox12*^*−/−*^ were higher than in WT mice, while *Alox15*^*−/−*^ joints were lower, although none were significantly different to WT. (Fig. 5D, E). Cartilage and bone erosion was significantly higher in *Alox12*^*−/−*^ mice at day 3 compared to *Alox15*^*−/−*^ (Fig. 5F). Overall, these data indicate that both strains appear to have a somewhat worsened joint phenotype than WT mice, but importantly, in contrast to its role in coagulation, *Alox12* does not drive joint disease in AIA.

### Synovial 12-HETE-PE is increased during AIA development, but generated by Alox15, not Alox12

Next, the profile of eoxPL in knee joints was analyzed to determine their temporal generation and biochemical origin. Basal 12- and 15-HETE eoxPL levels in pooled synovial tissues were similar for all strains (Fig. 5G, H, Supplemental Fig. S7). On induction of AIA in WT mice, these HETE-PEs significantly increased in joints on day 10 in WT and *Alox12*^*−/−*^, but not *Alox15*^*−/−*^ joints, confirming their enzymatic origin. Overall, in knee joints, the dominant source of 12-HETE-PEs generated during AIA induction was *Alox15*, directly contrasting with *Alox12* in the circulation.

### Alox15 deletion reduced generation of multiple oxylipins in synovial tissue during AIA development

**Fig. 6:**
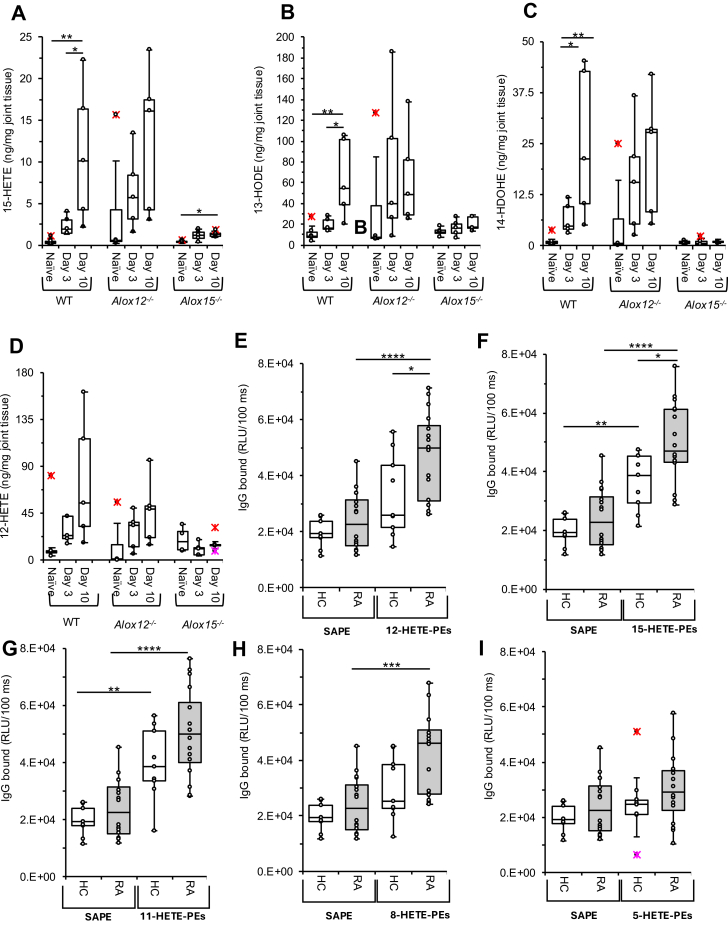
Abundant oxylipins from *Alox15* are generated during AIA development in mice, while circulating IgG recognizing HETE-PEs are increased in RA. Panels A-D: Generation of 15-HETE, 13-HODE, 14-HDOHE, and 12-HETE during AIA is dependent on Alox15. Oxylipins were quantified using LC/MS/MS, as outlined in Methods. Joints were collected and pooled for WT (n = 6, 6 joints/n), *Alox12*^*−/−*^ (n = 4, 6 joints/n), and *Alox15*^*−/−*^ (n = 5, 6 joints/n) naïve mice, as well as on days 3 and 10 of AIA development (n = 5, 3–4 joints/n). Data were analyzed using one-way ANOVA and Tukey’s multiple comparisons tests, comparing within genotypes (∗*P* < 0.05, ∗∗*P* < 0.01). Panels E-I: IgG directed against 12-,15- and 8-HETE-PEs are significantly increased in early RA patients compared to healthy controls. IgG levels against 12-, 15-, 11-, 8- and 5-HETE-PEs were determined as outlined in Methods and compared to IgG levels against SAPE (the same levels in all figures) in serum from HC (n = 9) and RA (n = 16) as described in Methods. Data were analyzed using two-way ANOVA and Tukey’s multiple comparisons tests (∗*P* < 0.05, ∗∗*P* < 0.01, ∗∗∗*P* < 0.001, ∗∗∗∗*P* < 0.0001). AIA, antigen-induced arthritis; HDOHE, hydroxydocosahexaenoic acid; HETE, hydroxyeicosatetraenoic acid; HODE, hydroxyoctadecadienoic acid; PE, phosphatidylethanolamine; RA, rheumatoid arthritis.

Free oxylipins were next analyzed in synovial tissue from WT, *Alox15*^*−/−*^ and *Alox12*^*−/−*^ mice following arthritis induction. During AIA, WT joints showed substantial elevations in 15-HETE, 13-HODE, and 14-HDOHE, and a trend towards an increase for 12-HETE (Fig. 6A–D). This increase was lacking in *Alox15*^*−/−*^ mice indicating 12/15-LOX as their origin (Fig. 6A–D). The full oxylipin dataset is presented in Supplemental Fig. S8. Overall, these data show that *Alox15* is the primary source of joint-derived 12- and 15-HETE in AIA-driven synovitis. These data contrast with blood, where *Alox12* was the primary source of abundant oxylipins during AIA induction, and is fully consistent with the enzymatic origin of eoxPL detected in blood and joints.

### Serum from early RA patients have elevated anti-HETE-PE IgG immunoreactivity

To test whether the thrombotic tendency in human RA might be similarly linked with platelet eoxPL, anti-HETE-PE IgG was measured in serum from a human cohort. Here, RA patients had been diagnosed ∼1 year before sampling, representing early disease (28). Only serum was available, allowing analysis of anti-HETE-PE IgG, but not TATs or eoxPL which require plasma or live cells. The IgG response to SAPE was not different between RA and HC serum, however, RA serum was more immunoreactive towards most HETE-PEs than HC, with 12- and 15-HETE-PEs being significantly higher in patients (Fig. 6E–I). Overall, these data indicate that early RA patients have a higher antigenic response to several HETE-PEs, in particular those from platelets, than healthy controls, suggesting higher exposure to these lipids in vivo.

## Discussion

RA patients are at higher risk of thrombosis, often leading to mortality (3, 4, 54). Here, we show that platelet *Alox12*, an enzyme that generates procoagulant lipids, is required for driving coagulation activity in genetically-altered mice with AIA (9, 12, 31). Importantly, since generation of platelet eoxPL, and elevations in SAA and TATs were entirely dependent on IL-6, a direct link between inflammatory signaling and downstream platelet activation is indicated. Using a human cohort we also found evidence for higher exposure to eoxPL in early RA. Further work is required to determine the complex regulation of thrombotic risk in human RA, including the generation and roles of eoxPL and whether their formation is mechanistically linked with the recently shown association of IL-6 targeted therapies (e.g., tocilizumab) with reduced coagulation markers in patients (5).

IL-6 increases platelet biogenesis from megakaryocytes through mechanisms involving interleukin-3, stem cell factor and thrombopoietin, generated by the liver. These young platelets are considered primed and hyper-responsive, as they are more sensitive to thrombin (reviewed in (55)). Separately, elevated circulating thrombopoeitin can directly prime mature platelets for aggregation in the circulation (55). Here, platelet 12-HETE-PE was elevated in the blood of AIA mice, without activation of platelets or increased platelet numbers. This suggests hyperactivation rather than increased biogenesis is responsible. Further studies are required to determine if this relates to higher expression of 12-LOX in these platelets or their megakaryocyte precursors, as well as the role of priming/activation.

Herein, we tested coagulation activity in the AIA model with mouse strains that develop distinct synovial pathotypes similar to human disease (31, 33, 46, 47, 56, 57). As our human cohorts did not have pathotype information, future studies will determine if the recently observed impact of anti-IL6-R therapy on coagulation markers (5) stratifies with these, in particular whether the fibroblast-rich form of RA that maps to *Il6ra*^*−/−*^ mice is less impacted by thrombosis as predicted by our study. Whether IL-6 targeted therapies differentially impact thrombotic risk in patients stratified into pathotypes remains to be tested.

We found that the increase in HETE-PEs seen in WT and *Il27ra*^*−/−*^ mice with AIA was mainly driven by *Alox12*-derived 12-HETE-PEs, indicating platelet involvement. This agrees with a previous study showing that generation of eoxPL in ex vivo clotting whole blood from healthy WT mice is mainly *Alox12*-dependent, with no contribution of the leukocyte isoform *Alox15* (22). Furthermore, mice lacking *Alox12* did not show elevated TATs in AIA, mechanistically linking coagulation activation (58) in vivo with this LOX isoform. This is fully in agreement with our previous study showing that i.v. injection of platelet-derived eoxPL acutely raise TAT levels in WT mice (12). It is also consistent with observations that administration of platelet-derived eoxPL directly into mouse tail tissue significantly shortens bleeding time either in WT or in mice lacking *Alox12* (12). Platelet involvement in AIA was further implicated by elevations in 15- and 11-HETE-PEs, generated by platelet COX-1. Importantly, *Alox12*^*−/−*^ mice demonstrated normal coagulation basally, indicating that the lack of TAT elevation was not due to reduced levels of factor activities. This is fully in line with the defect being at the level of procoagulant membranes instead. In contrast the lack of involvement of *Alox15* was intriguing. While previous studies have shown that this isoform can contribute to coagulation in circulating eosinophils in healthy mice (45), it appears that inflammatory induction of thrombosis via IL-6 targets platelets primarily rather than white cells, as evidenced by the pattern of eoxPL as well as impact of genetic deletion of *Alox* isoforms. Relating to therapeutic approaches, this indicates that the platelet (rather than leukocyte) procoagulant membrane would be the primary target. Further work is required to determine the direct impact of therapies targeting IL-6 on the procoagulant membrane, and how much this contributes to recent findings of reduced coagulation activity in patients on these drugs (5). Whether eoxPL act externally or throughout the membrane is currently unknown. While we can determine PE and PS externalization using LC/MS/MS, the levels of eoxPL are orders of magnitude lower in the platelet membrane and if we assume up to 7% is externalized (similar to PE/PS (59)), then their detection on the outside would be below the limit of detection for our assay. Relating to coagulation, this action of eoxPL is not dependent on hydrolysis, but requires the intact eoxPL to be present in the membrane.

Both WT and *Il27ra*^*−/−*^ mice develop leukocyte-driven synovitis and systemic inflammation, as evidenced by increased circulating SAA (47). Alongside this, they showed significantly increased TAT complexes and sP-selectin at day 10 and day 3, respectively. On the other hand, *Il6ra*^*−/−*^ mice did not demonstrate increases in blood cell eoxPL, SAA, sP-selectin, or TAT complexes. These mirror clinical findings in human patients treated with tocilizumab, an antibody against IL6-R, that controls inflammation by inhibiting the expression of acute phase reactants, including fibrinogen (5). Furthermore, IL-6 was previously shown to increase TATs in human plasma (60). Taken with our findings, this reveals that IL-6 directly drives eoxPL generation and downstream coagulation in AIA.

In contrast to our finding that platelet 12-LOX is exclusively responsible for blood cell eoxPL and TAT elevations, the leukocyte isoform generated eoxPL in joints during AIA. EoxPL can induce an antioxidative response (61) and increase clearance of apoptotic cells (62), and oxylipins such as 12-HETE, 15-HETE and 13-HODE, which were significantly reduced in synovial tissue on *Alox15* deletion, are also known PPARγ ligands (63). This suggests that 12/15-LOX maybe somewhat protective in synovitis, consistent with results from other mouse models of arthritis, through mechanisms that could involve both eoxPL and free oxylipins (64). Importantly, the data show that the two LOX isoforms play distinct tissue-specific roles in AIA with only 12-LOX involved in thrombotic risk. Neither LOX-deficient strain showed any reduction in SAA, indicating that inflammation acts upstream of eoxPL/TAT generation in AIA, consistent with the proposed role of IL-6.

In human disease, elevated autoantibody levels in early RA indicated exposure to circulating eoxPL, similar to the mouse model. In agreement, elevated eoxPL generation along with their IgG were previously shown in another prothrombotic disorder, APS (12). Although plasma was not available in the Leiden cohort for TAT analysis, thrombin activation markers are known to be significantly elevated in early RA patients with diagnosis of <24 months (5). This supports the idea that early RA is associated with both increased coagulation and eoxPL generation in humans. Whether anti-eoxPL IgG are protective or damaging in the context of autoimmunity-associated thrombosis is not known.

Despite increased thrombosis risk in RA, clinical guidelines do not currently address long-term prophylactic anticoagulation, with patients following the same rules as the general population (65, 66). In murine AIA, increased eoxPL, SAA, and TATs were completely dependent on IL-6, while in vitro SAA induces TF expression and activity (67). This suggests that biological medicines or small molecule inhibitors that target IL-6 signaling may reduce thrombosis in patients both through reducing TF as well as eoxPL. In conclusion, platelet 12-LOX is a central contributor to thrombotic risk in AIA, in an IL-6-dependent manner. In early human RA, a role for platelet 12-LOX in contributing to thrombotic risk is also suggested. These data indicate that reducing inflammation, particularly by targeting IL-6, may also suppress platelet reactivity to reduce thrombosis risk in RA.

### Study limitations

While the AIA model was chosen due to its relevance to human disease, we acknowledge that a murine model may not fully recapitulate the complexity of human RA. For example, this is a relatively short term model and it may be more relevant to early, rather than long established disease. Further studies are required to establish mechanisms of thrombosis risk in established RA, noting that the role of platelets may be different in that context. A further limitation relates to patient numbers which limited our ability to examine for potential impact of patient demographics and medication use.

## Data availability

All processed data are included in a supplemental file, while raw data are available on reasonable request.

## Supplemental data

This article contains supplemental data (22, 25, 29, 33, 47, 47, 68, 69, 70, 71, 72, 73, 74, 75).

## Conflict of interest

The contents of this article do not represent the views of the Department of Veterans Affairs or the United States Government. The content is solely the responsibility of the authors and does not necessarily represent the official views of the National Institutes of Health. E. C. has received research grants and honoraria from Abbvie, Alfasigma, Bio-Cancer, Biocon, Biogen, Chugai Pharma, Eli Lilly, Fresenius Kai, Galapagos, Gedeon Richter, Gilead, Inmedix, Janssen, Pfizer, Sanofi, UCB, and Viatris. S. A. J. has received funding support from Hoffman-La Roche, GlaxoSmithKline, Ferring Pharmaceuticals, Meastag Therapeutics, and NovImmune. S. A. J. has acted as an advisory consultant for Roche, Chugai Pharmaceuticals, NovImmune SA, Genentech, Sanofi Regeneron, Johnson & Johnson, Janssen Pharmaceuticals, Eleven Biotherapeutics, and Mab Design. All other authors declare that they have no conflicts of interest with the contents of this article.
