# Supplementary material for: Interleukin-6 stimulates platelet 12-lipoxygenase to drive coagulation in inflammatory arthritis
Source: J Lipid Res. 2026 Jun 29;67(8):101093. doi: 10.1016/j.jlr.2026.101093 (PMC13425847; doi:10.1016/j.jlr.2026.101093)

Supplementary Methods and Results:

Interleukin-6 stimulates platelet 12-lipoxygenase to drive coagulation in inflammatory arthritis.

Daniela O Costa[1], Stuart T.O Hughes[1], Robert H. Jenkins[1], Ana Cardus Figueras[1], Majd B Protty[1], Victoria J Tyrrell[1], Ali A Hajeyah[1], Gareth W Jones[2], James J Burston[1], Beth Morgan[1], Federica Monaco[1], David Hill[2], Aisling S Morrin[1], Carol Guy[1], Alice Bacon[3], Martin Giera[4], Rene E M Toes[3], P Vince Jenkins[5], Peter W Collins[1,6], Ernest Choy[1], Simon A. Jones[1], Valerie B O'Donnell[1]

[1] Systems Immunity Research Institute, Division of Infection and Immunity, School of Medicine, Cardiff University, Cardiff, CF14 4XN, UK

[2] School of Cellular and Molecular Medicine, University of Bristol, Bristol, United Kingdom.

[3] Department of Rheumatology, Leiden University Medical Center, Leiden, Netherlands.

[4] Center for Proteomics and Metabolomics, Leiden University Medical Center, Leiden, Netherlands

[5] Haematology Department, University Hospital of Wales, Cardiff, CF14 4XW, UK.

[6] University Hospital of Wales, Cardiff, CF14 4XW, UK.

Supplementary Methods

Murine antigen-induced arthritis (AIA), choice of model.
Beyond the reasons provided in Results, additional reasons for choosing AIA include ethical as well as biological considerations as follows. Ethically, AIA shows almost 100% incidence, reducing the number of animals needed, and is classed by the Home Office as a moderate protocol, meaning reduced discomfort to animals than severe models. Biologically, AIA allows for study of two distinct stages of disease (days 3, 10). AIA at these timepoints has been extensively characterized over many years *(1-5)* allowing study of how thrombotic risk develops and progresses in direct comparison with joint disease. Unlike other models, AIA can be induced in C57BL/6 mice as susceptibility is not limited to major histocompatibility complex (MHC) class II*(6)*. This allows the use of genetically-altered strains such as *Alox12*$^{-/-}$ and *Alox15*$^{-/-}$ which are on C57BL/6 background only. Joint swelling is an indicator of synovitis onset, resolving 4-5 days after intra-articular antigen administration. However, it is on its own a relatively poor indicator of overall disease activity, with histological assessments instead being required to measure the longer-term features of synovitis. As examples of this, synovial inflammation, exudate and pannus formation are all still present at day 10 in AIA, along with increased cartilage and bone erosion in wild-type mice at day 10 versus day 3 *(1,2,4,5,7)*.

Mouse Blood Collection. Mouse blood collection and processing were performed as previously described*(8)*. Whole blood was collected via cardiac puncture, preloaded with 100 ml of an anticoagulant mixture consisting of sodium citrate 3.8% (9:1, v/v) and 0.1 mg/ml corn trypsin inhibitor (Haematologic Technologies Inc., USA). Collected blood was immediately centrifuged at 3000 g for 5 minutes at room temperature, followed by plasma and whole blood cell isolation, which was all conducted on the same day. Blood cells were processed for lipidomics and then extracts frozen at -80 °until LC/MS/MS analysis. Mouse samples were collected over a 2-year time period then analysed as a single batch using LC/MS/MS.

Plasma was frozen at -80 °until analyzed for thrombin/antithrombin (TAT), prothrombin time (PT), D-dimers, serum amyloid A (SAA) as outlined below.

TAT complexes. TAT complexes were quantified in plasma using murine TAT ELISA Kit, as per the manufacturer's instructions (ab137994, Abcam, UK). Plasma samples were diluted at 1:100 before incubation with a TAT complex specific antibody. TAT complex specific biotinylated detection antibody was then added, followed by Streptavidin-Peroxidase conjugate. Chromogen Substrate was then added and left to react for 20 min before adding Stop Solution. Absorbance was immediately read on a microplate reader (CLARIOstar Plus, BMG Labtech) at 450 nm and values were corrected for background by subtracting readings at 570 nm. All samples and standards were analyzed in duplicate.

D-dimers. D-Dimers were analyzed using mouse D-Dimer, D2D ELISA Kit as per manufacturer's instructions (CSB-E13584m, Cusabio). Plasma samples were diluted at 1:500, before adding to each well. After 2h incubation, 100 µl biotin-conjugated antibody specific for D-dimers was added. This was followed by the addition of 100 µl avidin conjugated horseradish peroxidase. Subsequently, 90 µl TMB substrate was added. After incubation with substrate, color development was stopped using a stop solution and optical density read immediately on a microplate reader (CLARIOstar Plus, BMG Labtech) at 450 nm with the background of 570 nm subtracted. All samples and standards were analyzed in duplicate.

Serum Amyloid A. SAA in plasma was determined using a mouse SAA ELISA Kit as per the manufacturer's instructions (ab215090, Abcam, UK). Plasma samples were diluted 1:1000 before adding to each well. This was followed by the addition of antibody cocktail. After a 1-hour incubation, TMB Development Solution was added to each well. Stop solution was then added before reading absorbance at 450 nm on a microplate reader (CLARIOstar Plus, BMG Labtech). All samples and standards were analyzed in duplicate.

Soluble P-selectin. sP-selectin was analyzed using a mouse sP-selectin ELISA KIT as per the manufacturer's instructions (EMSELP, ThermoFisher, UK). Plasma samples were diluted 1:1000 before adding to each well. Samples and standards were incubated for 2.5h at room temperature, followed by incubation with biotin conjugate for 1h. The Streptavidin-HRP solution was then added and incubated for 45 min before the addition of 100 µl of TMB substrate. Once color development, the

reaction was stopped with spot solution and absorption read at 450 nm on a microplate reader (CLARIOstar Plus, BMG Labtech). All samples and standards were analyzed in duplicate.

Prothrombin time. Prothrombin time was measured in plasma using a coagulation analyzer (Amelung KC 10). Firstly, plasma was warmed in a water bath at 37 °C for 5 min. Plasma (40 µl) was then added to plastic cuvettes with a magnetic bead and incubated at 37 °C for 5 minutes. Next, 100 µl RecombiPlasTin 2G reagent (Werfen) was added, which promotes clot formation. The forming clot entangles the magnetic bead, resulting in the rotation of the bead within the cuvette. The time between the addition of RecombiPlasTin 2G reagent and the termination of the electromagnetic coupling represents designated prothrombin time, which is measured in seconds. Samples were analyzed in duplicate.

Platelet counts in mice. Mouse platelets were isolated from whole blood immediately after cardiac puncture with a needle containing 150 µl ACD [2.5% (w/v) trisodium citrate, 1.5% (w/v) citric acid, and 100 mM Glucose]. Blood was then added to an Eppendorf containing 150 µl of 3.8 % sodium citrate, followed by the addition of 300 µl Tyrode's buffer [145 mM NaCl, 12 mM $NaHCO_3$2.95 mM KCl, 1 mM $MgCl_2$, 10 mM HEPES and 5 mM glucose] (1:9 v/v). Blood was centrifuged (200 g, 5 mins, 22°C), followed by the collection of the supernatant as PRP, and the addition of 400 µl of Tyrode's buffer and recentrifuged (200 g, 2 mins, 22°C). The supernatant was recovered followed by a second addition of 400 µl of Tyrode's buffer and recentrifuged (500 g, 5 mins, 22°C). The remaining plasma was removed and pellet was resuspended in Tyrode's buffer. Platelets were counted with a hemocytometer.

Murine Sample Collection for Basal Coagulation Measurements. Male and female mice aged 12-14wks were anesthetized using 5% isoflurane and whole blood was collected via cardiac puncture into 3.8% sodium citrate at a ratio of 9:1. All blood samples were tested or processed to generate plasma and frozen within 30 minutes of collection. To generate plasma murine whole blood as described above was centrifuged at 3,000 x g for 10 minutes and the plasma layer was collected. Plasma samples were flash frozen in liquid nitrogen and stored at -80°C until use.

Prothrombin Time for Basal Coagulation Measurements. Prothrombin time (PT) was determined for murine plasma samples using a Merlin Medical MC 10 Plus ball coagulometer. Reagents and plasma samples were pre-heated to 37°C before testing. For PT measurements, 100 µl of HemosIL RecombiPlasTin 2G (Werfen, UK) was added to 50 µl of plasma and the time to clot was detected automatically. Where clotting failed to occur, the times were recorded as the maximum values of 120 seconds for PT.

Coagulation parameters using ROTEM assay. For ROTEM analysis of coagulation 50µl of activation mix containing Dade Innovin (B4212-50, Sysmex, UK) and $CaCl_2$ was added to the cup of a Werfen ROTEM Delta. 300 µl of mouse whole blood was then added to give a final concentration of 0.6% (v/v) Innovin and 10 mM $CaCl_2$. For analysis of fibrinolysis, the activation mix additionally contained Hyphen BioMed recombinant human t-PA (RP007C, Quadratech Diagnostics Ltd, UK) at a final concentration of 30 nM. Testing was performed at 37°C.

Mouse whole blood cell lipid extraction. Whole blood cell pellets were resuspended in 1 ml antioxidant buffer [ice-cold DPBS, 100 µM diethylenetriaminepentaacetic acid, 100 µM butylated hydroxytoluene, 7.5 µM acetaminophen, pH 7.4], followed by the addition of 10 µl of $SnCl_2$ (100 mM) for 10 min. Subsequently, internal standards were added. For whole blood lipidomics, SPLASH® LIPIDOMIX® Mass Spec Standard (Avanti Polar Lipids, USA) was used as an IS mixture, containing 10 ng PE(15:0/18:1(*d7*)) and 284 ng PC(15:0/18:1(*d7*)) added per sample. For oxylipin analysis, the IS used were deuterated lipids of the same class as the analyzed lipids, namely 2.3 ng 13(S)-HODE-d4, 2.5 ng 5(S)-HETE-d8, 2.5 ng 12(S)-HETE-d8, 2.5 ng 15(S)-HETE-d8, 2.5 ng 20-HETE-d6, 2.6 ng Leukotriene B4-d4, 2.9 ng Resolvin D1-d5, 2.5 ng Prostaglandin E2-d4, 2.7 ng Prostaglandin D2-d4, 2.7 ng Prostaglandin F2α-d4, 2.8 ng Thromboxane B2-d4, 2.8 ng 11-dehydro Thromboxane B2-d4, 2.5 ng11(12)-EET-d11 (Cayman Chemical, UK), as previously described*(9)*. Briefly, samples were transferred to 10 ml glass vial containing 2.5 ml ice-cold methanol. Lipids were extracted by adding 1.25 ml chloroform to each sample followed by incubation on ice for 30 minutes. Then, 1.25 ml chloroform and 1.25 ml water were added, and vortexed. The samples were then centrifuged at 400 g for 5 minutes at 4 °C to obtain a biphasic solution. Lipids were recovered from the bottom chloroform layer, and an additional 2.5 ml chloroform was then added the process repeated. The chloroform layers were then pooled and dried using a RapidVap Vacuum, re-suspended in methanol, and stored at − 80 °C prior to analysis by LC/MS/MS.

Mouse synovial tissue lipid extraction. Synovial tissue was dissected from the joint, weighed and pooled to achieve at least 5 mg per sample. For naïve tissue, 6 joints were pooled per sample, while during AIA, due to joint enlargement, 3-4 were sufficient per sample. We have at least 4 separate pooled samples in each group, which together represent numbers of joints per condition ranging from 15-36. Samples were then transferred to an Eppendorf tube with 0.5 ml antioxidant buffer [phosphate buffered saline, 100 µM diethylenetriaminepentaacetic acid, 100 µM butylated hydroxytoluene, 7.5 µM acetaminophen, pH 7.4]. Synovial tissue samples were homogenized in a Bead Rupture Elite® (2 cycles at 5 m/s for 20 seconds). Tissue samples were then transferred to glass vials and the remaining tissue was washed out with a further 0.5 ml antioxidant buffer, followed by the addition of internal standards, as described for mouse whole blood cell lipid extraction. The reduction of hydroperoxides was achieved by the addition of 10 µl of $SnCl_2$ (100 mM), and incubation for 10 minutes on ice. Lipids were first extracted using an isopropanol/hexane method, by adding 2.5 ml hexane/isopropanol/acetic acid (30:20:2, v/v/v) extraction solution to each sample. After vortexing, 2.5 ml hexane was added, followed by another vortexing step. The separation of phases was achieved by centrifugation (400 g, 5 mins, 4 °C). The upper layer was recovered and transferred to new extraction vials. Another 2.5 ml hexane was added, followed by another round of vortexing and centrifugation. The upper phase was recovered and combined with the previously recovered layer. The remaining bottom layer was then extracted using the Bligh and Dyer method by adding 2.5ml methanol and 1.25 ml chloroform. Samples were vortexed before adding 1.25 ml chloroform and 1.25 ml water. Samples were vortexed and centrifugated (400 g, 5 mins, 4 °C), and bottom layers recovered and dried using a RapidVap Vacuum, before being resuspended in methanol. To remove possible contaminating particles such as bone or cartilage, the samples were further extracted, using a final isopropanol/hexane method, which by recovering the upper layer, keeps the sedimented contaminating particles on the bottom layer. For this, 2.5 ml hexane/isopropanol/acetic acid (30:20:2, v/v) extraction solution was added to the lipid extract diluted in 1 ml of water. Following vortexing, another 2.5ml hexane was added. Upper layer was recovered after centrifugation (400 g, 5 mins, 4 °C). The upper layer was combined with other hexane layers. This combined hexane layers were then dried. Lipids were re-suspended in methanol and stored at -80 °C prior to analysis by LC/MS/MS.

Mouse knee joint histology. The whole knee joints were recovered, and following the removal of skin, knees were placed into histology cassettes and fixed in 10 % formalin for three days. This was followed by a decalcification process, where the cassettes were incubated in decalcification buffer (10 % formic acid in water). The tissue was then processed using the HistoCore PEARL before being embedded into paraffin blocks through Arcadia H instruments (Leica Biosystems). Parasagittal serial sections of 6 µm were obtained via a Leica RM2235 rotary microtome. The knee parasagittal sections were stained with hematoxylin and eosin, along with Safranin O and Fast Green (Sigma-Aldrich). Sections were scored by at least two independent observers (Supplementary Table 3), blinded to the experimental groups, as previously described*(10)*, using a Leica DM 2000 microscope and Leica Application Suite v4.9 software.

Supplementary Results and Discussion

Platelet-derived oxylipins are elevated in AIA via IL-6 signaling. Free oxylipins, including precursors for HETE-PEs, were analyzed. Of 93 measured, only 14 were detected with the most abundant being 12-HETE (Supplementary Figure 4). WT and *Il27ra*$^{-/-}$ mice showed increased 12-HETE on days 3 and 10, while no elevation was observed in *Il6ra*$^{-/-}$mice (Figure 4). Other HETEs were present at lower concentrations, with only 11- and 15-HETE quantifiable (Supplementary Figure 4). 11-HETE displayed a similar pattern to 12-HETE, while 15-HETE peaked on day 10 in *Il27ra*$^{-/-}$*,* remaining relatively low in other strains/times (Supplementary Figure 4). Similar to 12-HETE, other oxylipins, namely 13-HODE (Supplementary Figure 4 C), 14-HDOHE, 9-HODE, 13-HOTrE and 10-HDOHE (Figure 4, Supplementary Figure 4), peaked at day 3 of AIA in WT mice, displaying a significant increase compared to *Il6ra*$^{-/-}$. In the case of *Il27ra*$^{-/-}$ mice, the peak of these oxylipins, with the exception of 9-HODE, occurred on day 10 (Supplementary Figure 4). 12-HETrE levels were significantly increased in WT mice upon AIA development, especially at day 3, while in *Il27ra*$^{-/-}$ and *Il6ra*$^{-/-}$ mice a significant increase was observed at day 10 of AIA development (Supplementary Figure 4 G). 12-HETrE has been shown to downregulate platelet activity*(11)*. Leukotrienes, PGs, TXB_2, and specialized pro-resolving mediators (SPM) were not reliably detected at either time point, in any strains or in naïve

samples. Since many of these can originate from either 12-LOX or COX-1, the data suggest a platelet activation signature in AIA blood, without a PG-associated pro-inflammatory response.

Generation of HETEs and other oxylipins by blood cells are largely dependent on Alox12. Next, the contribution of LOXs to blood cell oxylipin elevations seen in AIA were determined using *Alox15$^{-/-}$* and *Alox12$^{-/-}$* blood. Basally, *Alox15$^{-/-}$* blood cells contained elevated levels of 12-, 11- and 15-HETEs, as well as 14-HDOHE, 10-HDOHE and 9-HODE, compared to WT (Figure 4, Supplementary Figure 5). *Alox12$^{-/-}$* blood cells contained less 12-HETE, as well as other oxylipins basally (Supplementary Figure 8 A). The most striking differences were that following AIA induction, *Alox12$^{-/-}$* blood did not show elevations in several oxylipins that were increased in WT blood, including: 12-, 11-HETEs, HDOHEs and HODEs (Figure 4, Supplementary Figure 5), indicating that they were dependent on this isoform. In contrast, the increased levels of 13-HODE in *Alox15$^{-/-}$* blood, both basally and during AIA development was somewhat unexpected since 13-HODE can be generated by 12/15-LOX oxidation of linoleic acid (Supplementary Figure 8 G)*(12)*. This suggests that 13-HODE might be generated either non-enzymatically or by another enzyme following *Alox15* deletion. 12-HETrE also increased in AIA at day 3 (Supplementary Figure 5 F), and was completely dependent on *Alox12* (Supplementary figure 8 I), although its levels were quite low compared to 12-HETE. As before, the pattern corresponds with that of eoxPL seen following AIA induction. Although these data show that most oxylipins and eoxPL depend on *Alox12$^{-/-}$* in platelets, it's not always clear which arise directly from enzymatic oxidation versus other mechanisms including (i) indirect non-enzymatic oxidation due to a small amount of radicals exiting the 12-LOX active site during turnover, and (ii) downstream effects such as reduced secondary platelet activation which could impact COX-1. However, the data show that *Alox12*, but not *Alox15*, is required for the oxylipin elevations seen in blood cells in AIA.

Generation of oxylipins in joints. 5-HETE was significantly elevated in WT mice on day 3, but this was not observed in either LOX-deficient strain, however, levels of this lipid were very low overall (Supplementary Figure 8 C). PGE_2 and PGD2 variably increased in WT and both *Alox$^{-/-}$* strains during AIA induction, consistent with COX-2 induction (Supplementary Figure 8 E,F). Some other oxylipins were detected at very low concentrations, including after AIA induction (Supplementary Figure 8). 7,17-diHDOHE was detected in WT joints at day 10, however, the level was below the assay LOQ with insufficient quantities to allow chiral analysis for ResolvinD5 structure verification*(13)*. No other putative specialized pro-resolving candidates were detected at any timepoint.

References

1. M. A. Nowell, A. S. Williams, S. A. Carty, J. Scheller, A. J. Hayes, G. W. Jones, P. J. Richards, S. Slinn, M. Ernst, B. J. Jenkins, N. Topley, S. Rose-John, S. A. Jones, Therapeutic targeting of IL-6 trans signaling counteracts STAT3 control of experimental inflammatory arthritis. *Journal of immunology (Baltimore, Md. : 1950)* **182**, 613–622 (2009).
2. G. W. Jones, M. Bombardieri, C. J. Greenhill, L. McLeod, A. Nerviani, V. Rocher-Ros, A. Cardus, A. S. Williams, C. Pitzalis, B. J. Jenkins, S. A. Jones, Interleukin-27 inhibits ectopic lymphoid-like structure development in early inflammatory arthritis. *The Journal of experimental medicine* **212**, 1793–1802 (2015).
3. A. Derrac Soria, D. Hill, S. T. Hughes, R. N. Scott, A. Cardus Figueras, S. Dimonte, D. Costa, N.-N. Vinh, F. Monaco, R. H. Jenkins, X. Liu, M. J. Lewis, J. Twohig, C. Guy, B. C. Cossins, A. S. Morrin, R. Andrews, B. Szomolay, L. Fossati, M. A. Nowell, A. S. Williams, E. H. Choy, B. J. Jenkins, N. M. Williams, H. Yu, M. Kortylewski, S. J. Turner, T. Tiganis, C. Pitzalis, G. W. Jones, S. A. Jones (2025) Discrete cytokine signaling networks instruct distinct synovial pathotypes in inflammatory arthritis. (Cold Spring Harbor Laboratory), pp 2025.2011.2001.686006–682025.686011.686001.686006.
4. F. Rivellese, D. Mauro, A. Nerviani, S. Pagani, L. Fossati-Jimack, T. Messemaker, F. A. S. Kurreeman, R. E. M. Toes, A. Ramming, S. Rauber, G. Schett, G. W. Jones, S. A. Jones, F. W. Rossi, A. De Paulis, G. Marone, M. E. M. El Shikh, F. Humby, C. Pitzalis, Mast cells in early rheumatoid arthritis associate with disease severity and support B cell autoantibody production. *Annals of the Rheumatic Diseases* **77**, 1773–1781 (2018).

5. J. P. Twohig, A. Cardus Figueras, R. Andrews, F. Wiede, B. C. Cossins, A. Derrac Soria, M. J. Lewis, M. J. Townsend, D. Millrine, J. Li, D. G. Hill, J. Uceda Fernandez, X. Liu, B. Szomolay, C. J. Pepper, P. R. Taylor, C. Pitzalis, T. Tiganis, N. M. Williams, G. W. Jones, S. A. Jones, Activation of naïve CD4 + T cells re-tunes STAT1 signaling to deliver unique cytokine responses in memory CD4 + T cells. *Nature Immunology* **20**, 458–470 (2019).
6. N. Choudhary, L. K. Bhatt, K. S. Prabhavalkar, Experimental animal models for rheumatoid arthritis. **40**, 193–200 (2018).
7. M. M. N. Wendt, M. C. de Oliveira, G. B. Franco-Salla, L. S. Castro, Â. V. Parizotto, F. M. Souza Silva, M. R. M. Natali, C. A. Bersani-Amado, A. Bracht, J. F. Comar, Fatty acids uptake and oxidation are increased in the liver of rats with adjuvant-induced arthritis. *Biochimica et Biophysica Acta (BBA) - Molecular Basis of Disease* **1865**, 696–707 (2019).
8. K. Allen-Redpath, M. Aldrovandi, S. N. Lauder, A. Gketsopoulou, V. J. Tyrrell, D. A. Slatter, R. Andrews, W. J. Watkins, G. Atkinson, E. McNeill, A. Gilfedder, M. Protty, J. Burston, S. R. C. Johnson, P. R. S. Rodrigues, D. O. Jones, R. Lee, A. Handa, K. Channon, S. Obaji, J. Alvarez-Jarreta, G. Krönke, J. Ackermann, P. V. Jenkins, P. W. Collins, V. B. O'Donnell, Phospholipid membranes drive abdominal aortic aneurysm development through stimulating coagulation factor activity. *Proceedings of the National Academy of Sciences* **116**, 8038–8047 (2019).
9. M. Misheva, K. Kotzamanis, L. C. Davies, V. J. Tyrrell, P. R. S. Rodrigues, G. A. Benavides, C. Hinz, R. C. Murphy, P. Kennedy, P. R. Taylor, M. Rosas, S. A. Jones, J. E. McLaren, S. Deshpande, R. Andrews, N. H. Schebb, M. A. Czubala, M. Gurney, M. Aldrovandi, S. W. Meckelmann, P. Ghazal, V. Darley-Usmar, D. A. White, V. B. O'Donnell, Oxylipin metabolism is controlled by mitochondrial β-oxidation during bacterial inflammation. *Nature Communications* **13**, 139 (2022).
10. G. W. Jones, D. G. Hill, K. Sime, A. S. Williams, In Vivo Models for Inflammatory Arthritis. *Methods Mol Biol* **1725**, 101–118 (2018).
11. B. E. Tourdot, R. Adili, Z. R. Isingizwe, M. Ebrahem, J. Cody Freedman, T. R. Holman, M. Holinstat, 12-HETrE inhibits platelet reactivity and thrombosis in part through the prostacyclin receptor. *Blood Advances* **1**, 1124–1124 (2017).
12. V. Vangaveti, B. T. Baune, R. L. Kennedy, Hydroxyoctadecadienoic acids: novel regulators of macrophage differentiation and atherogenesis. *Therapeutic advances in endocrinology and metabolism* **1**, 51–60 (2010).
13. N. H. Schebb, N. Kampschulte, G. Hagn, K. Plitzko, S. W. Meckelmann, S. Ghosh, R. Joshi, J. Kuligowski, D. Vuckovic, M. T. Botana, Á. Sánchez-Illana, F. Zandkarimi, A. Das, J. Yang, L. Schmidt, A. Checa, H. M. Roche, A. M. Armando, M. L. Edin, F. B. Lih, J. J. Aristizabal-Henao, S. Miyamoto, F. Giuffrida, A. Moussaieff, R. Domingues, M. Rothe, C. Hinz, U. S. Das, K. M. Rund, A. Y. Taha, R. K. Hofstetter, M. Werner, O. Werz, A. S. Kahnt, J. Bertrand-Michel, P. Le Faouder, R. Gurke, D. Thomas, F. Torta, I. Milic, I. H. K. Dias, C. M. Spickett, D. Biagini, T. Lomonaco, H. Idborg, J.-Y. Liu, M. Fedorova, D. A. Ford, A. Barden, T. A. Mori, P. D. Kennedy, K. Maxey, J. Ivanisevic, H. Gallart-Ayala, C. Gladine, M. Wenk, J.-M. Galano, T. Durand, K. D. Stark, C. Barbas, U. Garscha, S. L. Gelhaus, U. Ceglarek, N. Flamand, J. L. Griffin, R. Ahrends, M. Arita, D. C. Zeldin, F. J. Schopfer, O. Quehenberger, R. Julian, A. Nicolaou, I. A. Blair, M. P. Murphy, B. D. Hammock, B. Freeman, G. Liebisch, C. N. Serhan, H. C. Köfeler, P.-J. Jakobsson, D. Steinhilber, M. H. Gelb, M. Holčapek, R. Andrew, M. Giera, G. A. FitzGerald, R. C. Murphy, J. W. Newman, E. A. Dennis, K. Ekroos, G. L. Milne, M. A. Gijón, H. W. Vesper, C. E. Wheelock, V. B. O'Donnell, Technical recommendations for analyzing oxylipins by liquid chromatography–mass spectrometry. *Science Signaling* **18**, eadw1245 (2025).

Supplementary Figure Legends

Supplementary Figure 1. Immunization/priming of wild-type mice does not impact coagulation, inflammation or eoxPL generation while TATs and SAA are not impacted by *Alox15* deletion. *WT* mice 8-12 week old were primed using an i.p. injection of Pertussis toxin, and two s.c. injections of mBSA, but induction of arthritis was not performed. *Panel A. Priming does not impact TAT levels.* Plasma levels of TAT complexes were determined, as described in Methods, in WT naïve (n = 30)

and primed mice (n = 9). *Panel B. Priming does not impact D-dimer levels.* Plasma levels of D-dimers were determined, as described in Methods, in WT naïve (n = 27) and primed mice (n = 11). *Panel C. Prothrombin time is not impacted by priming.* Prothrombin time was determined in plasma, as described in Methods, in WT naïve (n = 10) and primed mice (n = 3). Data are represented as box and whisker plots. Data was analyzed using the Mann-Whitney test. *Panel D. TAT elevation in AIA is not dependent on Alox15.* TAT complexes were measured using ELISA. Plasma was collected on day 0 from WT naïve (n = 34), and *Alox15*$^{-/-}$ naïve (n = 11), as well as on days 3 and 10 of AIA development in *WT (n = 19 and 18, respectively),* and *Alox15*$^{-/-}$ (n = 7 for and n = 8, respectively) mice. Data were analyzed using Kruskal-Wallis test and Dunn's multiple comparisons tests, comparing within (**p<0.01, ***p<0.001). *Panel E. SAA elevation in AIA is not dependent on Alox15.* SAA was measured using ELISA. Plasma was collected on day 0 from WT naïve (n = 9), and *Alox15*$^{-/-}$ naïve (n = 6), as well as on days 3 and 10 of AIA development in WT (n = 15 and 11, respectively), and *Alox15*$^{-/-}$ (n = 8 and 7, respectively) mice. Data were analyzed using Kruskal-Wallis test and Dunn's multiple comparisons tests, comparing within (**p<0.01, ***p<0.001, ****p<0.0001). *Panels F-J. EoxPL generation is not significantly altered following immunization/priming alone. WT* mice, between 8-12 weeks old, were primed using an ip injection of Pertussis toxin, and two s.c. injection of mBSA, without the induction of arthritis. Whole blood was collected on day 0 from WT naïve (n = 23) and primed (n = 11) mice. Lipids from whole blood cell pellets were extracted as described in Methods and analyzed using LC/MS/MS and the sum of individual HETE-PEs positional isomers was calculated. Data is represented in box and whisker plots. Data were analyzed using Mann-Whitney test.

Supplementary Figure 2. ***Alox15* deletion doesn't prevent generation of most HETE-PEs in mouse blood cells during AIA development,** AIA was induced in 8-12 week old *WT and Alox15*$^{-/-}$ male mice as described in Methods, with whole blood collected on Days 3 and 10. HETE-PL were quantified as outlined in Methods using LC/MS/MS*.* Whole blood was collected from WT (n = 30) and *Alox15*$^{-/-}$ (n = 17), as well as on days 3 and 10 of AIA development *WT* (n = 23 and 18 respectively) and *Alox15*$^{-/-}$ (n = 8 for both days)*. Panel A. Heatmap showing generation of increased levels of eoxPL during AIA.* HETE-PEs were quantified as outlined in Methods using LC/MS/MS. Heatmaps show log10 concentration (ng/ml). *Panels B-F. Individual data on oxylipins showing impact of Alox15*$^{-/-}$. Data were analyzed using one-way ANOVA and Tukey's multiple comparisons tests, comparing within genotypes (*p<0.05, **p<0.01,*** p<0.001).

Supplementary Figure 3. Induction of AIA is not altered in *Il27ra*$^{-/-}$ or *Il6ra*$^{-/-}$ mice, while platelet count, D-dimers and PT are also unaffected by AIA, and a heatmap summarizes eoxPL data. *Panel A. Joint swelling confirms the induction of AIA in all strains.* Mice joint diameters of WT (n = 16), *Il27ra*$^{-/-}$(n = 12) and *Il6ra*$^{-/-}$(n = 20) mice were measured on day 0, before intra-articular injection, and on days 1, 2, 3, 7 and 10 after mBSA administration. Percentage swelling was calculated with a peak observed between days 2 and 3, confirming the induction of arthritis. *Panel B. Platelet count does not vary between mouse genotypes.* Platelets were isolated and counted from WT (n = 6), *Il27ra*$^{-/-}$ (n = 4), *Il6ra*$^{-/-}$ (n = 4), *Alox15*$^{-/-}$ (n = 4) and *Alox12*$^{-/-}$ (n = 4) mice. Data is represented as box and whisker plots. Data were analyzed using One-way ANOVA and Tukey's multiple comparison test. *Panel C. AIA model does not significantly alter platelet count.* Platelets from WT and *Il6ra*$^{-/-}$ mice were isolated and counted in control mice (n = 6 and n = 4, respectively), and at day 3 (n = 4 and n = 5, respectively) and day 10 (n = 5 and n = 8) of AIA model development. Data are represented as box and whisker plots. Data were analyzed using Two-way ANOVA and Tukey's multiple comparison test. *Panel D. AIA induction in WT, Il27ra*$^{-/-}$ *and Il6ra*$^{-/-}$ *does not alter D-Dimer levels.* D-dimers were measured using ELISA. Plasma was collected from WT naïve (n = 33), *Il27ra*$^{-/-}$ naïve (n = 3) and *Il6ra*$^{-/-}$ naïve (n = 3), as well as on days 3 and 10 of AIA development from WT (n = 25 and 23, respectively), *Il27ra*$^{-/-}$ (n= 9 for both days) and *Il6ra*$^{-/-}$ (n= 6 and 4, respectively) mice. Data were analyzed using the one-way ANOVA test and Tukey's multiple comparison test within genotypes. *Panel E. AIA induction does not alter prothrombin time.* Prothrombin time was determined as described in Methods. Plasma was collected from WT naïve (n = 12), *Il27ra*$^{-/-}$ naïve (n = 8) and *Il6ra*$^{-/-}$ naïve (n = 7), as well as on days 3 and 10 of AIA development in WT (n = 8 and 9, respectively), *Il27ra*$^{-/-}$ (n = 7 and 6, respectively) and

Il6ra$^{-/-}$ (n = 5 for both days) mice. Data were analyzed using one-way ANOVA and Tukey's multiple comparison test within genotypes. *Panel F. HETE-PEs increase during development of AIA, but not in Il6ra*$^{-/-}$ *mice.* HETE-PEs were quantified as outlined in Methods using LC/MS/MS. Heatmaps show log10 concentration values (ng/ml) for quantified HETE-PEs.

Supplementary Figure 4. Oxylipins are elevated in blood cells from WT and *Il27ra*$^{-/-}$ during AIA development, especially on day 10. AIA was induced in 8-12 week old *WT, Il27ra*$^{-/-}$ and *Il6ra*$^{-/-}$ male mice as described in Methods. *Panels A-F.* Whole blood was collected from WT (n = 25), *Il27ra*$^{-/-}$ naïve (n = 7) and *Il6ra*$^{-/-}$ (n = 10), as well as, on day 3 and 10 of AIA development in *WT* (n = 19 for day 3; n = 18 for day 10), *Il27ra*$^{-/-}$ (n = 9 for both days) and *Il6ra*$^{-/-}$ (n = 5 for both days) mice. Lipids from whole blood cell pellets were extracted as described in Methods and analyzed using LC/MS/MS for 13-HODE, 11- and 15-HETEs, 9-HODE, 13-HOTrE and 10-HDODE. Data were analyzed using one-way ANOVA and Tukey's multiple comparison test, comparing within genotypes (*p<0.05, **p<0.01, ***p<0.001, ****p<0.0001). *Panel G.* Lipids were extracted from whole blood cell pellets from WT (n = 16), *Il27ra*$^{-/-}$ (n = 7) and *IL6ra*$^{-/-}$ (n = 8), as well as, on day 3 and 10 of AIA development in *WT* (n = 18 for day 3; n = 10 for day 10), *Il27ra*$^{-/-}$ (n = 9 for day 3; n = 8 for day 10) and *Il6ra*$^{-/-}$ (n = 5 for day 3; n = 4 for day 10) mice and quantified as outlined in Methods for 12-HETrE. Data were analyzed using one-way ANOVA and Tukey's multiple comparison test, comparing within genotypes (*p<0.05, **p<0.01, ****p<0.0001). *Panel F. Oxylipins are increased during AIA development, but not in Il6ra*$^{-/-}$ mice. Oxylipins were quantified as outlined in Methods using LC/MS/MS. Heatmaps show log10 concentration (ng/ml).

Supplementary Figure 5. *Alox12* deletion prevents increases in free oxylipins in whole blood during AIA development. AIA was induced in 8-12-week old *WT, Alox12*$^{-/-}$ and *Alox15*$^{-/-}$ male mice as described in Methods. Whole blood was collected from WT naïve (n = 25), *Alox15* $^{-/-}$ naïve (n = 17) and *Alox12*$^{-/-}$ naïve (n = 5), as well as, on day 3 and 10 of AIA development in *WT* (n = 19 for day 3; n = 18 for day 10), *Alox15*$^{-/-}$ (n = 8 for both days) and *Alox12*$^{-/-}$ (n = 7 for both days) mice. *Panels A-F. Alox12 deletion decreases generation of 12-HETE, 14-HDODE, 9-HODE and 10-HDODE, while increasing 15-HETE during AIA development.* Lipids from whole blood cell pellets were extracted as described in Methods and analyzed using LC/MS/MS. 11- and 15-HETE, 14-HDODE, 9- and 13-HODEs and 10-HDODE were determined. Data is represented in box and whisker plots. Data were analyzed using one-way ANOVA and Tukey's multiple comparison test between genotypes (* p <0.05, ** p <0.01, *** p<0.001, **** p<0.0001). Panel I. *Alox12 deletion prevents the generation of 12-HETrE in blood during acute AIA development.* Lipids from whole blood cell pellets were extracted as described in Methods and analyzed using LC/MS/MS. Whole blood was collected from WT naïve (n = 16), *Alox15* $^{-/-}$ naïve (n = 8) and *Alox12*$^{-/-}$ naïve (n = 5), as well as, on day 3 and 10 of AIA development in *WT* (n = 18), *Alox15*$^{-/-}$ (n = 7) and *Alox12*$^{-/-}$ (n = 6) mice. Data were analyzed using one-way ANOVA and Tukey's multiple comparison test, comparing within genotypes (* p <0.05, ** p <0.01, *** p<0.001, **** p<0.0001). *Panel G. Alox12 is responsible for oxylipin generation in blood cells during AIA.* Oxylipins were quantified as outlined in Methods using LC/MS/MS. Heatmaps show log10 concentration values (ng/ml).

Supplementary Figure 6. AIA development in both *Alox15*$^{-/-}$ *and Alox12*$^{-/-}$ mice results in a worse phenotype while antibody response to mBSA is similar for WT, *Alox15*$^{-/-}$ mice and *Alox12*$^{-/-}$ mice. *Panel A. Antibodies against mBSA in mouse plasma.* Specific antibody titres against mBSA were determined using ELISA in WT and *Alox15*$^{-/-}$ mice plasma on day 10 post arthritis induction. Data represents mean ± SEM (n = 4) and statistical analysis was performed using a student t-test. *Panel B. Histological staining.* AIA was induced in 9-12-week old *WT, Alox12*$^{-/-}$ and *Alox15*$^{-/-}$ male mice as

described in Methods, with synovial tissue collected on Days 3 and 10. Knee joints were also collected from WT, *Alox12*$^{-/-}$ and *Alox15*$^{-/-}$ naïve mice for histological staining and assessment, as described in Methods. Representative images of haematoxylin, fast green and safranin O staining of WT (top), *Alox12*$^{-/-}$ (middle) and *Alox15*$^{-/-}$ (bottom) mouse knee joints as controls (left), and at days 3 (centre) and 10 (right) of AIA development.

Supplementary Figure 7. *Alox15* deletion results in *an overall* reduction of oxPLs in synovial tissue during AIA development. AIA was induced in 9-12 week old WT, *Alox12*$^{-/-}$ and *Alox15*$^{-/-}$ male mice as described in Methods, with knee joints collected from WT naïve (n = 6), *Alox15*$^{-/-}$ naïve (n = 4) and *Alox12*$^{-/-}$ naïve (n = 4), as well as, on day 3 and 10 of AIA development from *WT* (n = 5), *Alox15*$^{-/-}$ (n = 5) and *Alox12*$^{-/-}$ (n = 5) mice. Lipids from pooled synovial tissue were extracted as described in Methods and analyzed using LC/MS/MS. *Panel A. Alox15 is responsible for the generation of increased 12- and 15-HETE-PE species during AIA development in synovial tissue.* Heatmaps shows log10 concentration values [ng/mg (wet tissue)] for HETE-PEs species. *Panel B. Deletion of Alox15 or Alox12 does not alter the generation of 11-HETE-PEs during AIA development in synovial tissue.* The sum of 11-HETE-PEs was quantified as outlined in Methods using LC/MS/MS. Data is represented in box and whisker plot. Data were analyzed using one-way way ANOVA and Tukey's multiple comparison test, comparing within genotypes.

Supplementary Figure 8. *O*xylipins generation in synovial tissue during AIA development. AIA was induced in 9-12 week old WT, *Alox12*$^{-/-}$ and *Alox15*$^{-/-}$ male mice as described in Methods, with knee joints collected from WT (n = 6), *Alox15*$^{-/-}$ (n = 4) and *Alox12*$^{-/-}$ (n = 4) naïve mice, as well as, on day 3 and 10 of AIA development in *WT* (n = 5), *Alox15*$^{-/-}$ (n = 5) and *Alox12*$^{-/-}$ (n = 5) mice. Lipids from pooled synovial tissue were extracted as described in Methods and analyzed using LC/MS/MS. *Panel A. Oxylipin profiles during AIA development.* Heatmap shows log10 concentration values [ng/mg (wet tissue)]. *Panel B-F.* The concentration of oxylipins in synovial tissue (ng/mg tissue) were determined as described in Methods and analyzed using LC/MS/MS. Data were analyzed using one-way way ANOVA and Tukey's multiple comparison test, comparing within genotypes (*p<0.05, **p <0.01).

Supplementary Figure 1

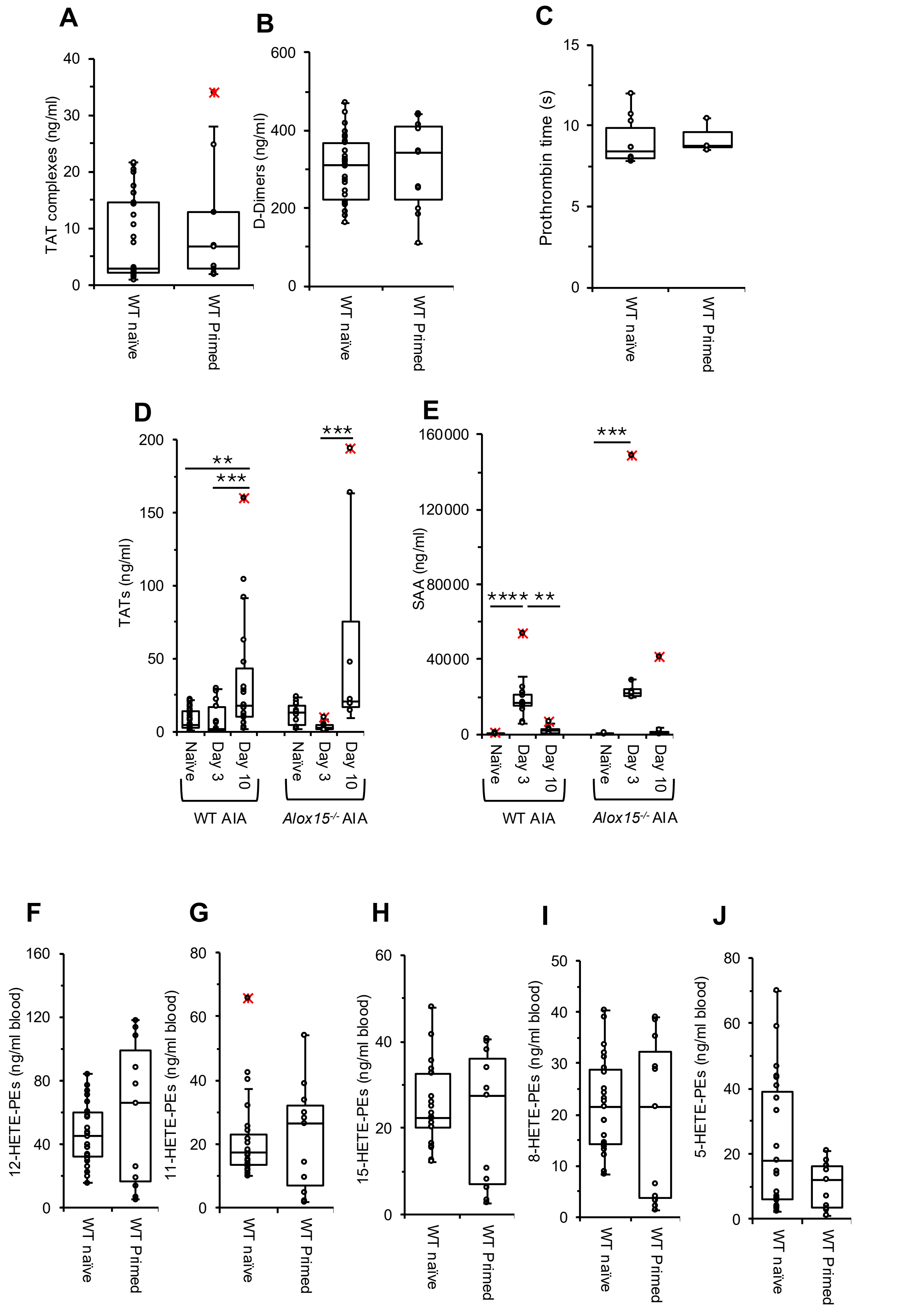

Supplementary Figure 2

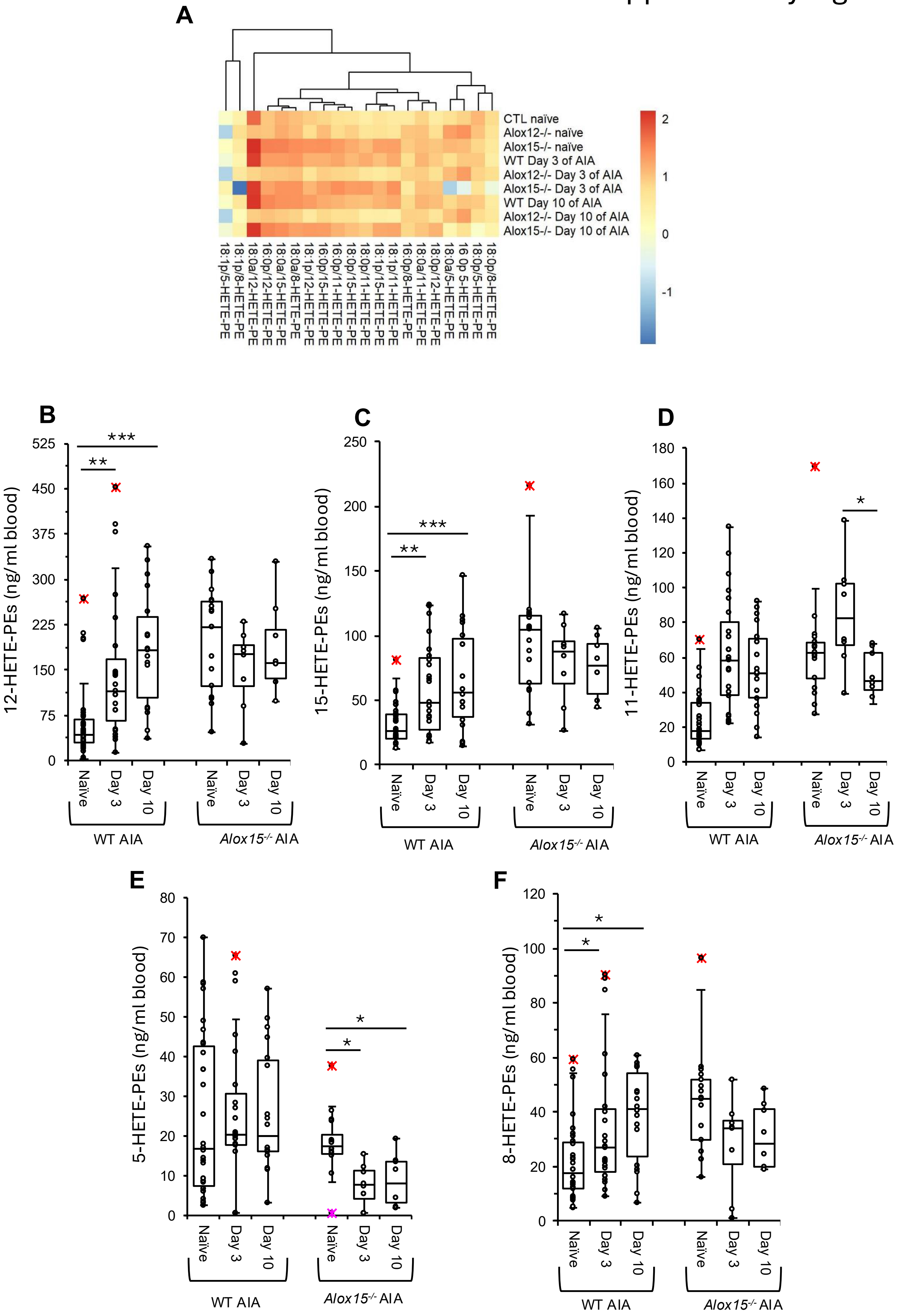

Supplementary Figure 3

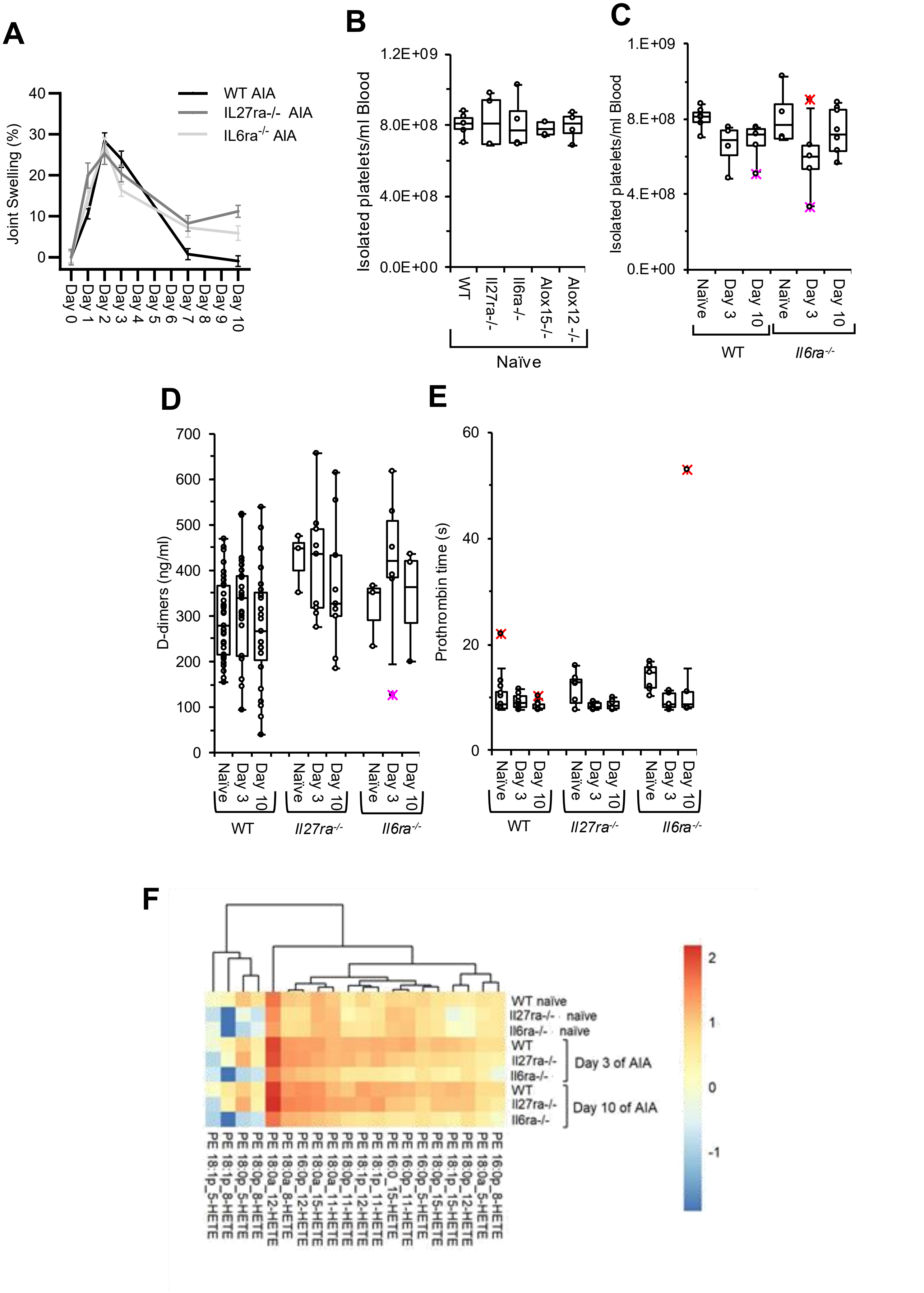

Supplementary Figure 4

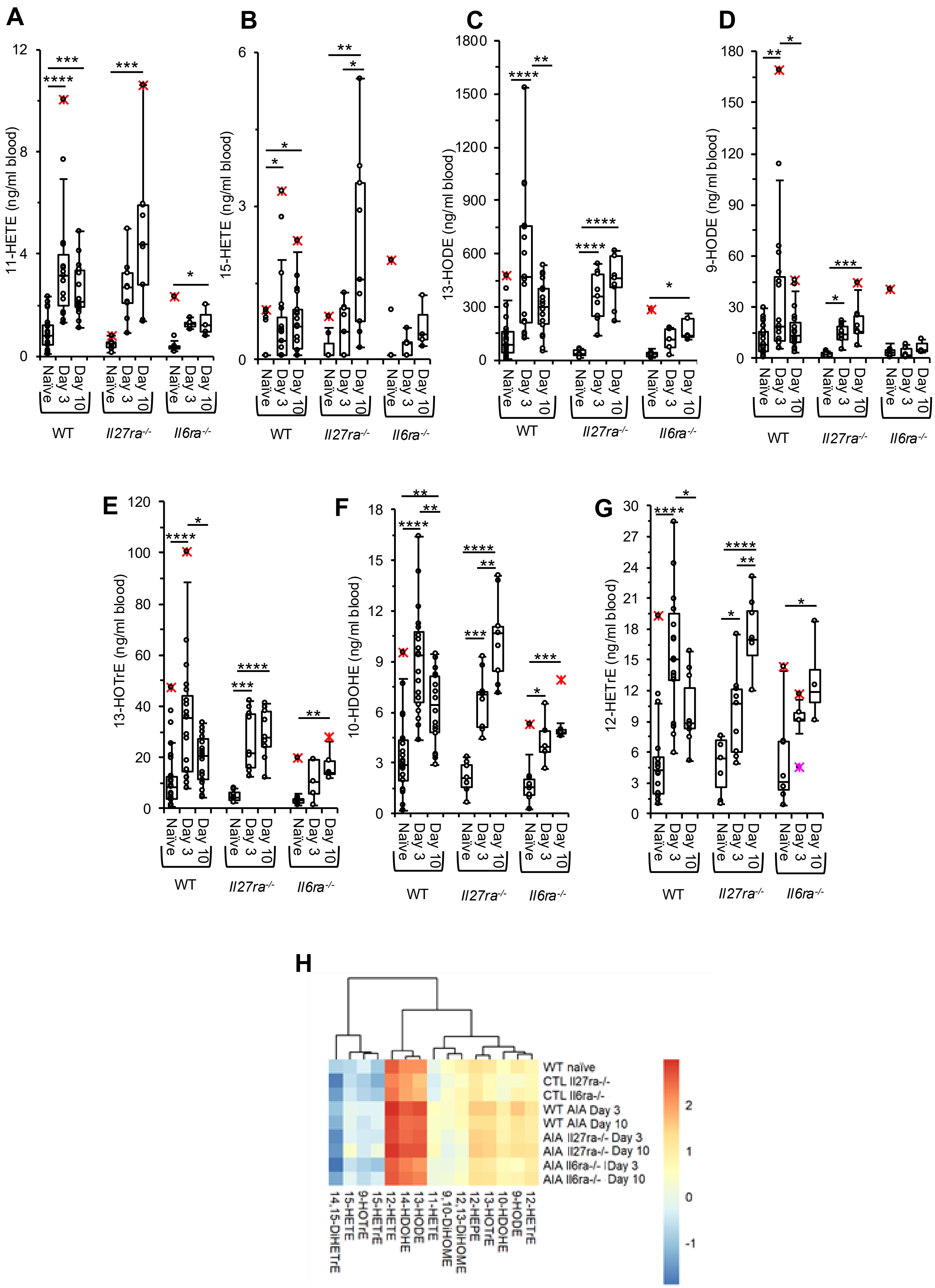

Supplementary Figure 5

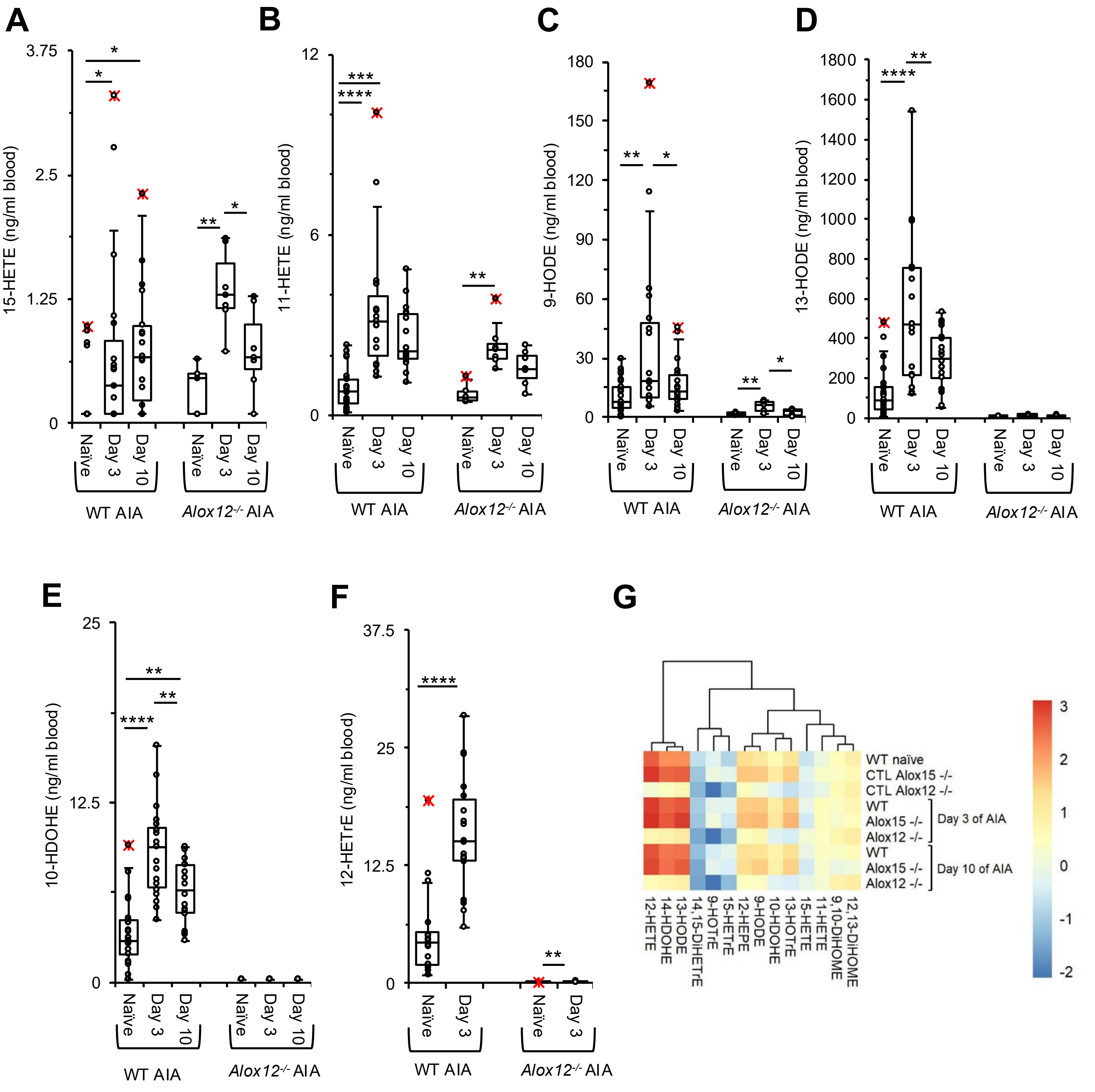

Supplementary Figure 6

A

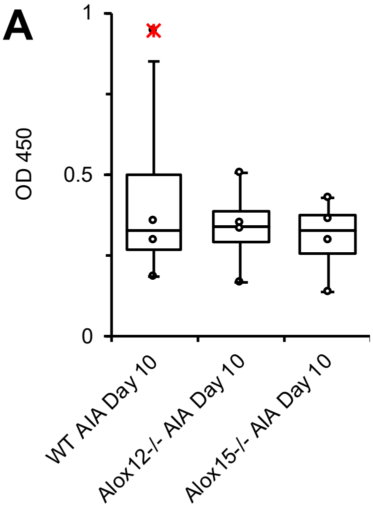

B

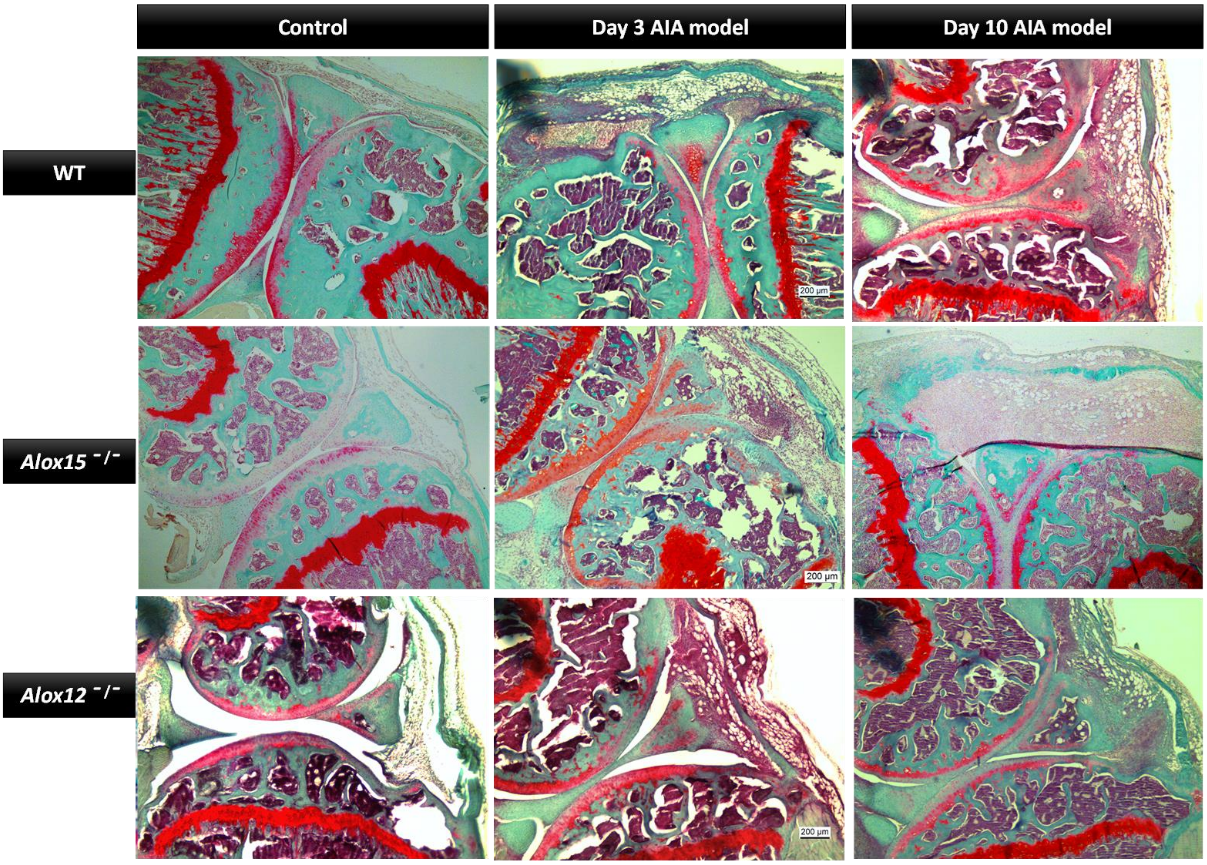

Supplementary Figure 7

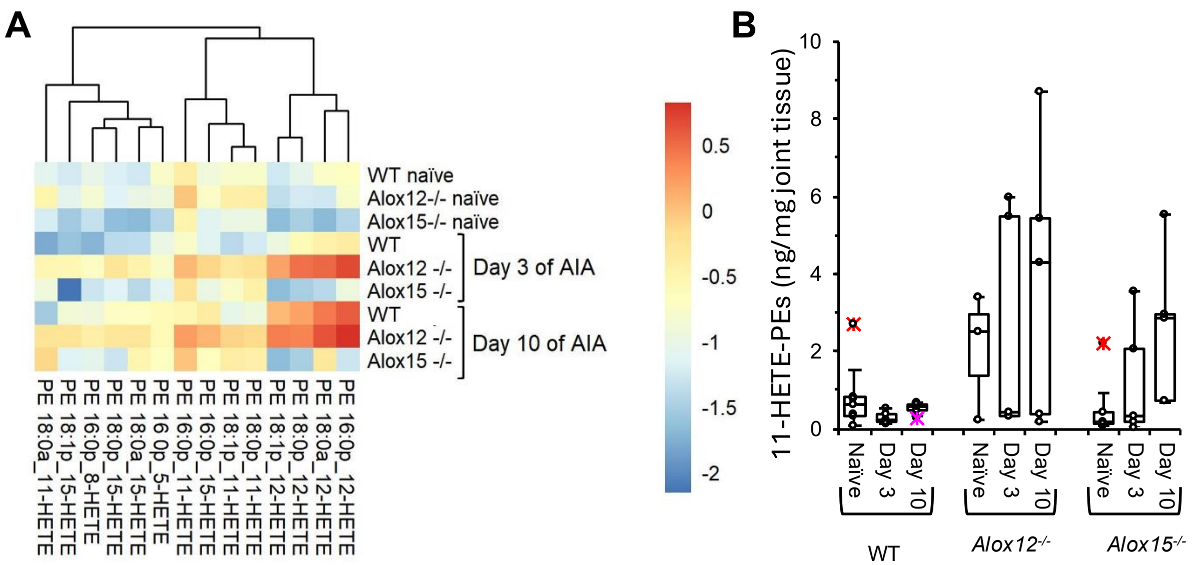

Supplementary Figure 8

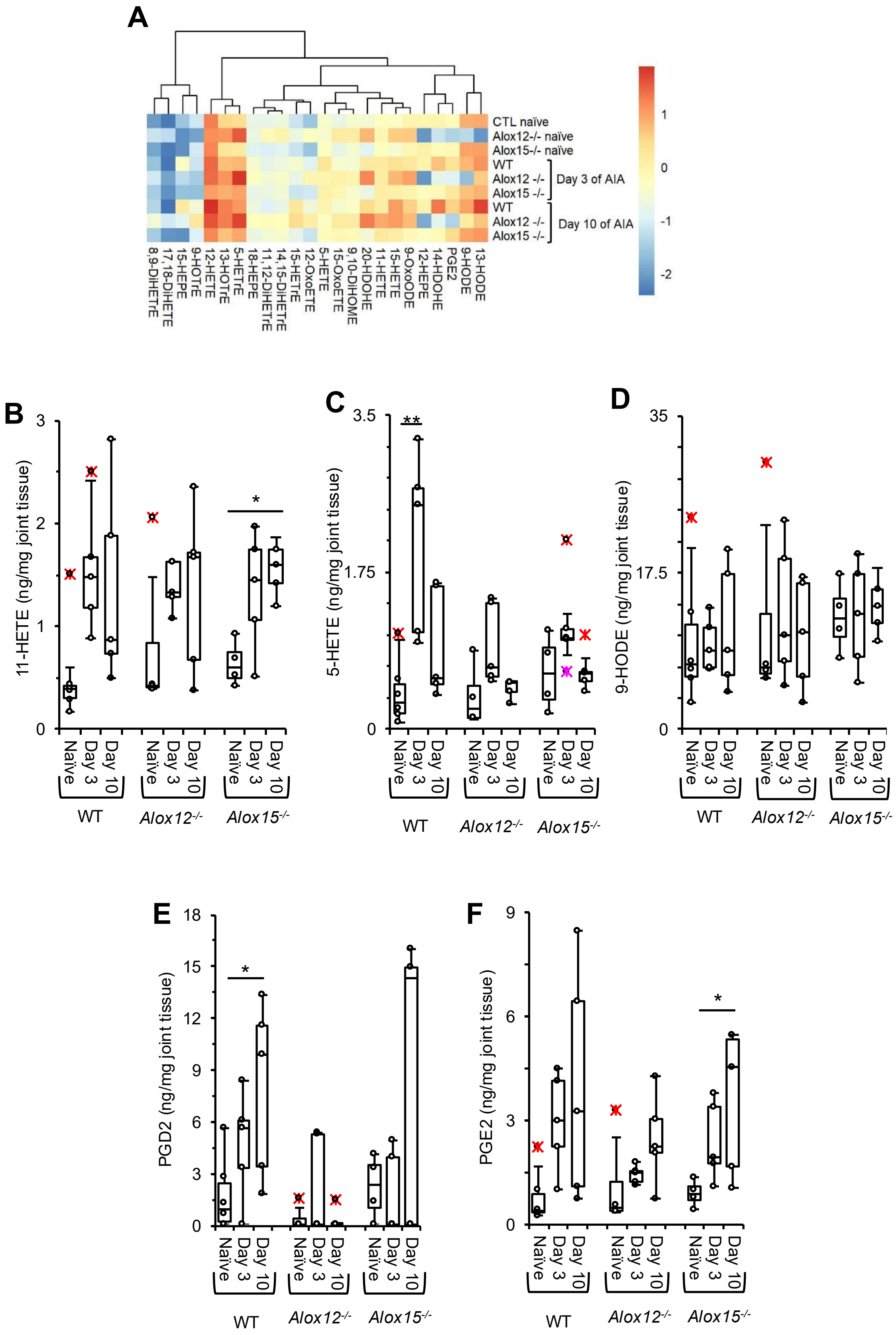

Supplement: Supplemental Materials [file mmc2.pdf]
